# MRPL3 enhances mitochondrial function via the TOMM40/PGC-1α/TFAM axis to drive early recurrence in hepatocellular carcinoma

**DOI:** 10.1038/s41419-026-08968-8

**Published:** 2026-06-09

**Authors:** Shubin Wang, Wuhan Yang, Teng Pan, Huiyan Deng, Zheng Wu, Xing Li, Xiaokuan Zhang, Lu Bai, Qi Feng, Huifang Wang, Lei Nie, Li Peng, Zhiyu Wang

**Affiliations:** 1 Department of General Medicine, The Fourth Hospital of Hebei Medical University, Shijiazhuang, PR China; 2 Department of Hepatobiliary Surgery, The Fourth Hospital of Hebei Medical University, Shijiazhuang, PR China; 3 Department of Oncology, Shijiazhuang People’s Hospital, Shijiazhuang, PR China; 4 Department of Pathology, The Fourth Hospital of Hebei Medical University, Shijiazhuang, PR China; 5 Department of Immuno-Oncology, The Fourth Hospital of Hebei Medical University, Shijiazhuang, PR China; 6 Department of Biochemistry and Molecular Biology, School of Basic Medicine, Hebei Medical University, Shijiazhuang, PR China

**Keywords:** Liver cancer, Prognostic markers

## Abstract

Early recurrence after curative resection remains a major obstacle to improving outcomes in hepatocellular carcinoma (HCC). Given the pivotal role of mitochondrial reprogramming in tumor progression, we investigated the contribution of mitochondrial ribosomal protein L3 (MRPL3) to postoperative early recurrence and its underlying mechanisms. Label-free quantitative proteomic profiling of HCC tissues identified MRPL3 as a recurrence-associated candidate. Its overexpression in HCC was confirmed by qRT-PCR, western blotting, and immunohistochemistry, and correlated with poor disease-free and overall survival. Functional assays demonstrated that MRPL3 enhances HCC cell proliferation, migration, and invasion both in vitro and in vivo by improving mitochondrial respiration, membrane potential, and ATP production while reducing reactive oxygen species levels. Mechanistically, MRPL3 associates with TOMM40, a core component of the mitochondrial outer membrane translocase complex, through its ΔR1 (1–174 aa) region and the ΔR2 (181–361 aa) domain of TOMM40, forming a protein complex that contributes to the activation of the PGC-1α/TFAM signaling pathway. Disruption of this axis abrogated the metabolic and oncogenic effects of MRPL3. Collectively, these findings identify MRPL3 as a key mitochondrial regulator that promotes metabolic reprogramming and drives early recurrence of HCC through the TOMM40/PGC-1α/TFAM axis, suggesting its potential as a prognostic biomarker and therapeutic target to prevent postoperative relapse.

**Figure:**
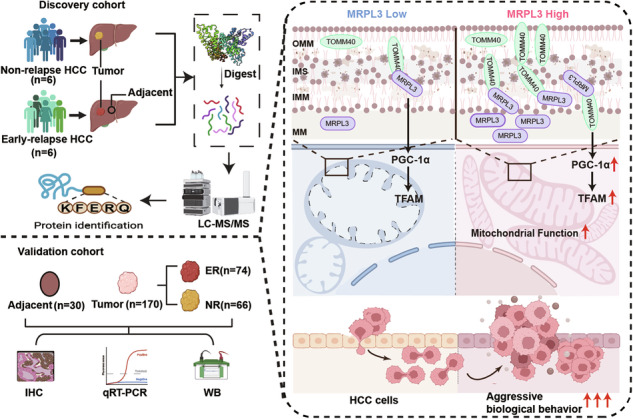

## Introduction

Hepatocellular carcinoma (HCC) constitutes approximately 75% of liver cancer cases, making it the sixth most frequently diagnosed malignancy globally [1]. Although curative resection remains the mainstay for early-stage HCC, the 5-year postoperative recurrence rate remains as high as 50–70%, leading to poor outcomes [2–4]. Notably, early recurrence within 2 years postoperatively represents roughly 70% of all recurrence events and markedly compromises survival and quality of life [5, 6]. Further, although numerous studies have investigated clinical predictors of early recurrence [7–10], the underlying molecular mechanisms remain poorly understood.

Since the proposal of the Warburg effect, metabolic reprogramming has emerged as a central theme in oncology [11]. Cancer cells often display mitochondrial dysfunction alongside enhanced glycolysis [12]; however, accumulating evidence indicates that tumors can rewire metabolism and rely on mitochondrial function to sustain rapid proliferation and migration under microenvironmental constraints[13, 14]. Oxidative phosphorylation (OXPHOS) contributes ~80% and ~83% of adenosine triphosphate (ATP) production in normal and cancer cells, respectively [15]. In HCC, mitochondrial alterations are tightly linked to malignant progression, including changes in OXPHOS activity, respiratory chain defects, ATP production, and imbalanced reactive oxygen species (ROS) [16–19]. These mitochondrial adaptations not only support metabolic plasticity but also modulate the immune tumor microenvironment and contribute to therapeutic resistance.

Mitochondrial ribosomes orchestrate the translation of 13 mitochondria-encoded proteins that are essential for mitochondrial function and energy homeostasis [20]. Mitochondrial ribosomal protein L3 (MRPL3), an essential part of the large (39S) subunit, is crucial for mitochondrial protein synthesis and the assembly and function of respiratory complexes. Recent studies have reported aberrant MRPL3 expression across multiple malignancies—including pancreatic ductal adenocarcinoma, HCC, lung adenocarcinoma, and prostate and breast cancers—and linked it to tumor metabolism, proliferation, and invasion/metastasis [21–25]. However, the role and mechanisms of MRPL3 in HCC, particularly in postoperative early recurrence, remain unclear. Investigating its impact on mitochondrial function, metabolic reprogramming, and early recurrence in HCC could offer new insights into disease progression and highlight a potential therapeutic target. Therefore, this study aimed to explore the role of MRPL3 in early HCC recurrence and its molecular mechanisms.

## Methods and materials

### Clinical specimens

Tumor and adjacent non-tumor tissues were obtained from patients with pathologically confirmed HCC who underwent curative hepatectomy at the Fourth Hospital of Hebei Medical University. Between January and December 2017, 42 tumor specimens and 33 paired adjacent tissues were collected, rapidly preserved in liquid nitrogen, and stored at −80 °C until analysis. Twelve tumors and three matched non-tumor samples were selected for label-free quantitative proteomic analysis (sample details in Supplementary Table 1). RNA was extracted from 30 frozen tumor-adjacent pairs for quantitative reverse transcription polymerase chain reaction (qRT-PCR) analysis, while protein extraction and western blotting were conducted on 24 randomly selected pairs to evaluate MRPL3 expression. In addition, a retrospective cohort of patients with HCC who underwent curative resection between January 2017 and December 2018 was analyzed. Formalin-fixed and paraffin-embedded (FFPE) tumor samples and corresponding clinical data were collected, with follow-up until November 20, 2023. All patients were pathologically confirmed as having HCC and had not received preoperative radiotherapy or chemotherapy. The evaluation of microvascular invasion (MVI) was conducted according to the “Evidence-based Practice Guidelines for the Pathological Diagnosis of Primary Liver Cancer (2015 Edition)” [26]. The study was approved by the hospital ethics committee (protocol 2023KS181), and written informed consent was obtained from all participants. No formal statistical method was used to predetermine the sample size for the clinical cohort. All eligible patients who met the predefined inclusion and exclusion criteria during the study period were consecutively enrolled.

### RNA extraction and qRT-PCR

Total RNA was isolated from human HCC tissues using TRIZOL reagent (Invitrogen, USA) and from cultured cells using a commercial RNA isolation Kit (Yeasen, China), respectively. One microgram of RNA was treated with DNase and reverse-transcribed into cDNA using the Hifair III First Strand cDNA Synthesis Kit (Yeasen, China). The resulting cDNA was diluted at a ratio of 1:5 and analyzed with Hieff UNICON Universal Blue qPCR SYBR Green Master Mix (Yeasen, China). Relative mRNA expression was calculated using GAPDH as the internal control by the comparative Ct (2^−ΔΔCt^) method. Primer sequences, synthesized by Generay Biotech (Shanghai, China), are detailed in Supplementary Table 2.

### Western blot analysis

Protein was extracted from tissue homogenates and cultured cells using RIPA buffer (NCM, WB2100) supplemented with protease inhibitors. Equal amounts of denatured protein (20–40ug) were separated via sodium dodecyl sulfate-polyacrylamide gel electrophoresis (SDS-PAGE). After blocking with 5% non-fat milk for 1.5 h, membranes were incubated with primary antibodies overnight at 4 °C. Horseradish peroxidase (HRP)-conjugated secondary antibodies were applied for 1 h at room temperature. Immunoreactive bands were visualized using an enhanced chemiluminescence kit (MCE, HY-K1005) and captured on a GE ImageQuant™ LAS 4000 imaging system. Supplementary Table 2 provides a comprehensive list of all antibodies used in this study, including vendor and catalog number.

### Immunohistochemistry (IHC) staining and scoring

Human HCC tissues and mouse xenograft tumors, both FFPE, were sectioned into 4-μm slices for IHC staining using the EnVision method on a Roche Benchmark GX automated IHC platform, following the manufacturer’s guidelines. MRPL3 expression was evaluated based on both the percentage of positively stained cells and staining intensity. The percentage of positive cells was scored as 1 (0–5%), 2 (6–25%), 3 (26–50%), or 4 ( > 50%). Staining intensity was categorized into three levels: 1, weak; 2, moderate; and 3, strong. The final IHC score was calculated as the product of the two parameters, with scores ≥8 considered high MRPL3 expression [27]. Two independent pathologists, blinded to clinical data, reviewed all slides.

### Cell lines and culture

Human HCC cell lines (HepG2, HuH-7, MHCC97-H, and SK-HEP-1) and HEK293T cells were sourced from the Cell Bank of the Chinese Academy of Sciences. The Mahlavu human HCC cell line and THLE-2 immortalized human hepatocyte line were obtained from Procell Life Science & Technology. All cell lines were authenticated by short tandem repeat (STR) profiling and tested negative for mycoplasma contamination before use. All cells were cultured in Dulbecco’s modified Eagle’s medium (DMEM) containing 10% fetal bovine serum and 1% penicillin/streptomycin at 37 °C in a humidified atmosphere with 5% CO₂.

### Transfection and generation of stable cell lines

Lentiviral vectors for MRPL3 knockdown and overexpression were developed and obtained from GeneChem in Shanghai, China. Cells were infected and then selected with 2 μg/mL puromycin for 2 weeks to create stable transfectants. SiRNAs targeting TOMM40 and mitochondrial transcription factor A (TFAM) were sourced from HanHeng Biotechnology in Shanghai, China. Further, transfections were conducted in six-well plates using RNAFit reagent (HANBIO, HB-RF-1000) following the manufacturer’s instructions. Cells were harvested 36–48 h later for subsequent experiments. All sequences are detailed in Supplementary Table 2.

### Cell proliferation, migration, and invasion assays

Cell proliferation was assessed using the CCK-8 reagent (HANBIO, HB-CCK-8-500T) and colony formation assays. Cell migration was evaluated by the wound-healing assay, and wound closure was quantified after 24 h. Transwell assays were performed to examine cell migration and invasion. For migration assays, cells were seeded into the upper chambers, while for invasion assays, the upper chambers were pre-coated with Matrigel.

### Animal models

Six-week-old male BALB/c nude mice were purchased from Beijing Huafukang Bioscience Co., Ltd. (China). All animal experiments were approved by the Institutional Animal Care and Use Committee of the Fourth Hospital of Hebei Medical University and conducted in accordance with relevant guidelines. Humane endpoints were predefined, and the maximal tumor burden permitted by the committee was not exceeded. Sample size was estimated based on preliminary experiments and published studies using similar xenograft models. No animals were excluded from the analysis.

For the subcutaneous xenograft model, two independent experiments were conducted. In the main-figure experiment, mice were randomly assigned to the vector or oeMRPL3 groups (*n* = 6 per group) using simple randomization and subcutaneously injected in the flank with HuH-7 cells stably expressing the corresponding constructs. In the supplementary experiment, mice were randomly assigned to the shNC or shMRPL3 groups (*n* = 4 per group) using the same method and injected in the same manner. Tumor growth was measured every 3 days, and tumor volume was calculated as length × (width²) × 0.5. On day 30, mice were humanely euthanized, and tumors were excised, photographed alongside a ruler, and processed for subsequent analyses.

For the orthotopic liver tumor model, Mahlavu cells stably expressing Vector or oeMRPL3 were suspended in sterile PBS and injected subcapsularly into the left liver lobe under isoflurane inhalation anesthesia at 2 × 10^6^ cells in 40 μL per mouse. Mice were randomly divided into the indicated groups. For OXPHOS inhibition experiments, oligomycin treatment was initiated on day 7 after implantation. Oligomycin was administered by intraperitoneal injection at 1 mg/kg three times per week, and control mice received vehicle. Mice were sacrificed on day 21, and liver tissues were collected for blinded histological and immunohistochemical analyses.

### Transmission electron microscopy (TEM)

Cells were fixed in 2.5% glutaraldehyde at 4 °C overnight, then post-fixed in 1% osmium tetroxide at the same temperature, dehydrated through graded ethanol, infiltrated with graded acetone, and subsequently embedded. Ultrathin sections(≈50 nm) were stained with uranyl acetate and lead citrate, and examined on a Hitachi HT7800 electron microscope.

### Mitochondrial respiration

High-resolution respirometry was performed using an Oroboros O2k oxygraph. Cells were exposed sequentially to oligomycin, carbonyl cyanide-4-(trifluoromethoxy)phenylhydrazone (FCCP), rotenone, and antimycin A, with oxygen consumption measured after each addition. Values for basal and ATP-linked respiration, proton leakage, maximal respiration, spare capacity, and residual non-mitochondrial respiration were calculated to characterize mitochondrial function.

### Mitochondrial membrane potential and ROS measurements

The JC-1 Mitochondrial Membrane Potential Assay Kit (G1515-100T, Servicebio) was utilized to assess mitochondrial membrane potential, following the manufacturer’s instructions and analyzed by fluorescence microscopy. Cellular and mitochondrial ROS levels were assessed using the ROS Assay Kit (S0033S, Beyotime) and MitoSOX Red (Thermo Fisher), respectively. Fluorescence intensity was quantified via flow cytometry (CytoFLEX LX, Beckman Coulter).

### RNA sequencing (RNA-seq)

Total RNA from the oeMRPL3 and vector control groups was extracted using TRIZOL reagent. RNA-seq was performed by Sinotech Genomics (Shanghai, China) following standard protocols. Genes exhibiting differential expression were identified based on the criteria of *P* < 0.05 and an absolute log₂ fold change of at least 1. Functional enrichment analyses of differentially expressed genes were conducted using the R packages ClusterProfiler and ReactomePA, and results were visualized using ggplot2 and enrichplot.

### Co-immunoprecipitation (Co-IP)

Cells were lysed in NP-40 buffer containing protease inhibitors, and equal protein amounts were prepared. Lysates were incubated with specified antibodies at 4 °C for 4 h, followed by the addition of protein A/G agarose beads (Thermo Fisher, USA) and overnight rotation at 4 °C. Immune complexes were pelleted, washed five times using PBS with Tween 20, resuspended in 1× SDS loading buffer, boiled, and analyzed via SDS-PAGE and western blotting.

### Immunoprecipitation coupled with liquid chromatography-tandem mass spectrometry (IP-LC-MS/MS) analysis

For IP-LC-MS/MS, Mahlavu and HuH-7 cells were lysed in IP buffer as described above. Protein (1 mg) was incubated with anti-MRPL3 antibody (5 μL) or control IgG for immunoprecipitation. Proteins were eluted using 5× loading buffer and separated via SDS-PAGE. Gels were stained using a mass spectrometry-compatible fast silver staining kit (C500021, Sangon Biotech). Protein bands of interest were excised, diced, and analyzed using LC-MS/MS by GeneChem (Shanghai, China) to identify MRPL3-interacting proteins.

### Immunofluorescence (IF) staining

Cells were seeded on coverslips, fixed with paraformaldehyde, and permeabilized with Triton X-100. After blocking with goat serum, cells were incubated with primary antibodies at 4 °C overnight, followed by fluorophore-conjugated secondary antibodies for 2 h. Nuclei were counterstained with DAPI and imaged using a Leica confocal microscope.

### Proximity ligation assay (PLA)

Cells were seeded on glass coverslips in 24-well plates, fixed with 4% paraformaldehyde, permeabilized with 0.2% Triton X-100, and blocked using Naveni Block. The in situ proximity ligation assay (PLA) was performed with the NaveniFlex Cell Red kit (Navinci, Sweden) according to the manufacturer’s instructions. Nuclei were stained with DAPI, and protein–protein interaction signals were observed using a Leica confocal laser scanning microscope (Germany).

### Statistical analysis

All analyses were performed with GraphPad Prism 10.0 and R 4.1.3. Data are expressed as mean ± SD. Each experiment was performed at least three times independently. Differences between two groups were evaluated by a two-tailed Student’s *t* test, while multiple groups were compared using one-way or two-way ANOVA followed by Tukey’s test. Kaplan–Meier survival curves were compared using the log-rank test. Prognostic factors were assessed by Cox regression, and a nomogram was constructed based on multivariate results. Model performance was evaluated using calibration plots and time-dependent AUC analysis. Significance levels were denoted as *P < 0.05, **P < 0.01, ***P < 0.001; ns, not significant.

## Results

### Label-free quantitative proteomic analysis of postoperative early-recurrent versus non-recurrent HCC

**Fig. 1:**
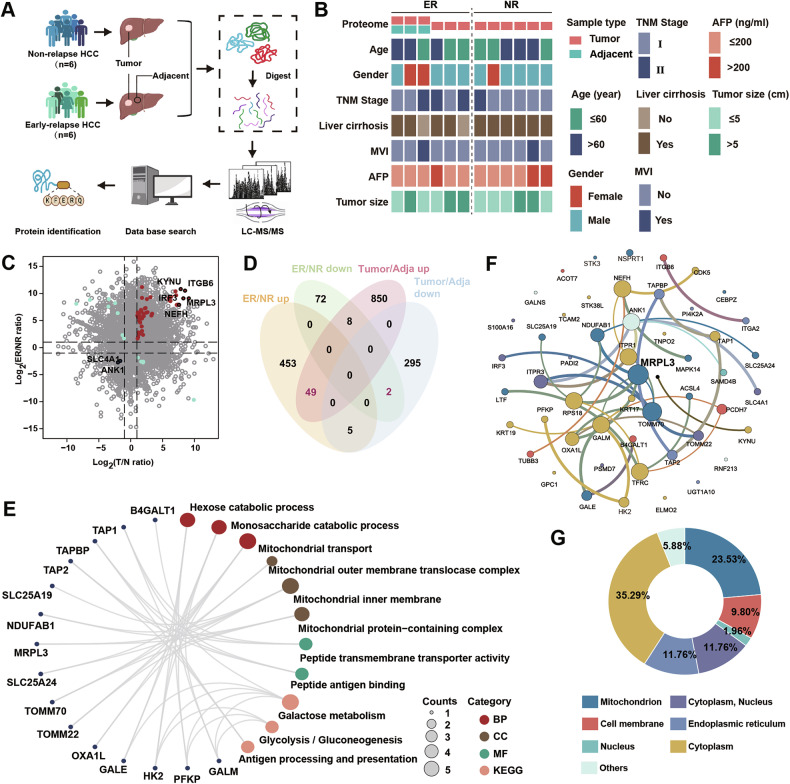
Label-free quantitative proteomic profiling of early-recurrent versus non-recurrent HCC. **A** Study workflow and sample allocation (number per group). **B** Clinicopathologic summary of 12 patients with HCC, among whom three adjacent non-tumor tissues were obtained from early-recurrent cases. **C** Quadrant scatter plot of protein expression: x-axis, tumor vs. adjacent differential; y-axis, early-recurrent tumors (ER) vs. non-recurrent tumors (NR); colored dots denote significantly altered proteins (DEPs, *p* < 0.05). **D** Venn diagram showing the overlap of significant DEPs between ER and NR. **E** GO and KEGG enrichment of upregulated DEPs in ER. **F** Protein–protein interaction (PPI) network of DEPs. **G** Subcellular localization and distribution of DEPs. HCC hepatocellular carcinoma, DEPs differentially expressed proteins.

To delineate the molecular basis of postoperative early recurrence in HCC, we performed label-free quantitative proteomics on tumor tissues from six patients with early recurrence (ER) and six without recurrence (NR), together with three matched adjacent non-tumor tissues (Fig. 1A). Figure 1B and Supplementary Table 1 presents the clinicopathological data alongside follow-up information. In total, 6,856 proteins were identified by mass spectrometry (Supplementary Fig. 1A), with principal component analysis clearly separating tumor and adjacent tissues (Supplementary Fig. 1B) and density distribution of log10-transformed intensities showing highly comparable signal profiles across samples, indicating good data quality and effective normalization (Supplementary Fig. 1C). Compared with adjacent tissues, tumors exhibited 1209 differentially expressed proteins (DEPs), including 907 upregulated; compared with NR tumors, ER tumors harbored 589 DEPs, of which 507 were upregulated (Fig. 1C). The intersection of upregulated DEPs between the two comparisons yielded 49 proteins (Fig. 1D). Gene Ontology/Kyoto Encyclopedia of Gene and Genome enrichment analyses indicated that upregulated DEPs in ER were significantly enriched in mitochondrial pathways (Fig. 1E). Further, a protein–protein interaction (PPI) network highlighted functional connectivity among the upregulated DEPs (Fig. 1F), and subcellular localization analysis placed most of them in the cytosol and mitochondria (Fig. 1G). Collectively, these data implicate mitochondrial dysfunction in the postoperative early recurrence of HCC.

### MRPL3 is highly expressed in HCC and correlates with early recurrence following surgery

**Fig. 2:**
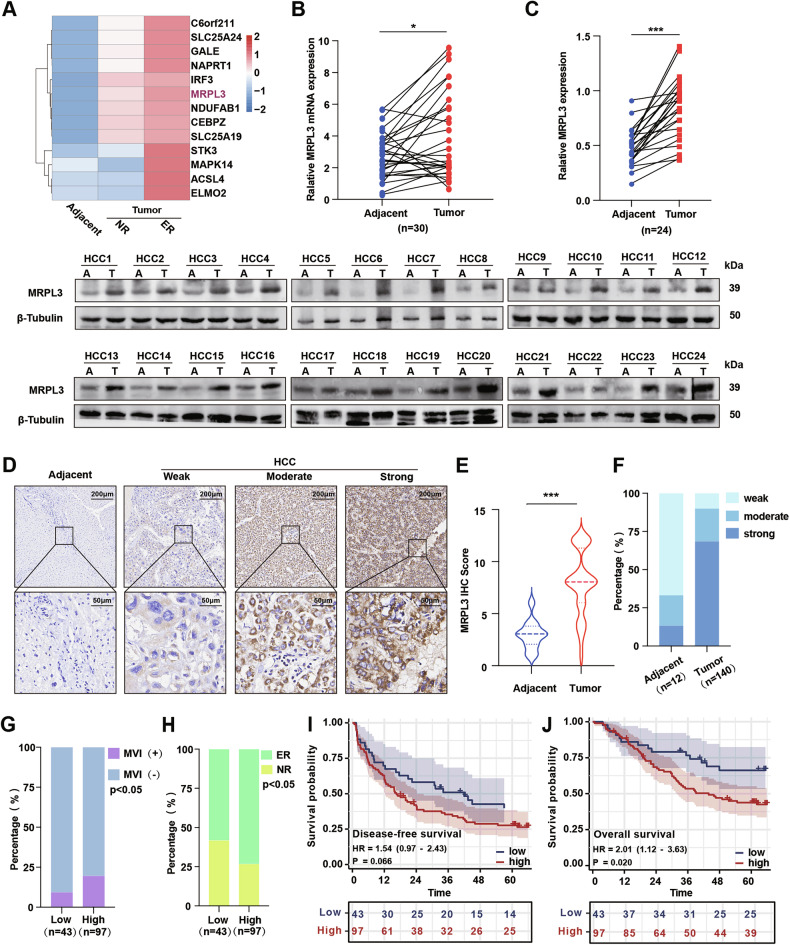
MRPL3 is overexpressed in HCC and correlates with early recurrence. **A** Heatmap of mitochondria-related proteins in early-recurrent tumors (ER), non-recurrent tumors (NR), and adjacent non-tumor tissues. Paired analyses showing significantly higher MRPL3 levels in tumors versus adjacent tissues at the mRNA (**B**, *n* = 30) and protein (**C**, *n* = 24) levels; paired t test. **D** Representative IHC images of MRPL3 in tumor and adjacent tissues. **E** Quantification of MRPL3 protein by IHC score in tumors (*n* = 140) versus adjacent tissues (*n* = 12); Mann–Whitney U test. **F** Proportions of high/medium/low MRPL3 expression in tumor and adjacent tissues; chi-square test. Correlations between MRPL3 expression (IHC score) and microvascular invasion (**G**) or early recurrence (**H**); Spearman’s rank correlation. Kaplan–Meier survival analyses comparing high- versus low-MRPL3 groups: disease-free survival (DFS, **I**; *P* = 0.066) and overall survival (OS, **J**; *P* = 0.02); log-rank test. Data are presented as the mean ± SD (*n* ≥ 3 unless stated). Significance: ns, not significant; *P < 0.05; **P < 0.01; ***P < 0.001. HCC hepatocellular carcinoma, IHC immunohistochemistry, SD standard deviation.

Given the potential importance of mitochondrial function in postoperative early recurrence, we examined mitochondrial-related DEPs across ER, NR, and matched adjacent tissues (Fig. 2A). Integration with the PPI network highlighted mitochondrial ribosomal protein MRPL3 as a putative driver of early recurrence. We further compared MRPL3 expression between HCC tumors and paired adjacent tissues: both qRT-PCR (*n* = 30) and western blotting (*n* = 24) demonstrated significantly higher MRPL3 mRNA and protein levels in tumors (Fig. 2B, C). IHC revealed predominant cytoplasmic localization of MRPL3 (Fig. 2D).

In a retrospective cohort study of 140 patients who underwent resection for HCC between January 2017 and December 2018, IHC scores and staining intensity were notably elevated in tumor tissues compared with adjacent non-tumor tissues (Fig. 2E, F; clinicopathological and follow-up data are shown in Fig. 1B and Supplementary Table 1). MRPL3 overexpression correlated with microvascular invasion (MVI) and early recurrence (Fig.2G, H and Supplementary Table 3). Kaplan–Meier analyses showed a trend toward poorer disease-free survival (DFS) and significantly worse overall survival (OS) in the high-MRPL3 group compared with the low-expression group (DFS, *p* = 0.066; OS, *p* = 0.02; Fig. 2I, J). Multivariate Cox regression analysis determined MRPL3 as an independent prognostic indicator for DFS and OS (Supplementary Tables 4, 5). LASSO regression further selected MRPL3 expression and American Joint Committee on Cancer (AJCC) stage as key prognostic variables for DFS and OS (Supplementary Fig. 1D, G), and nomograms integrating these factors exhibited good predictive and calibration performance (Supplementary Fig. 1E, F, H, I). MRPL3 was significantly overexpressed in HCC, with its elevated levels strongly linked to poor prognosis and early recurrence.

### MRPL3 enhances HCC proliferation, migration, and invasion

**Fig. 3:**
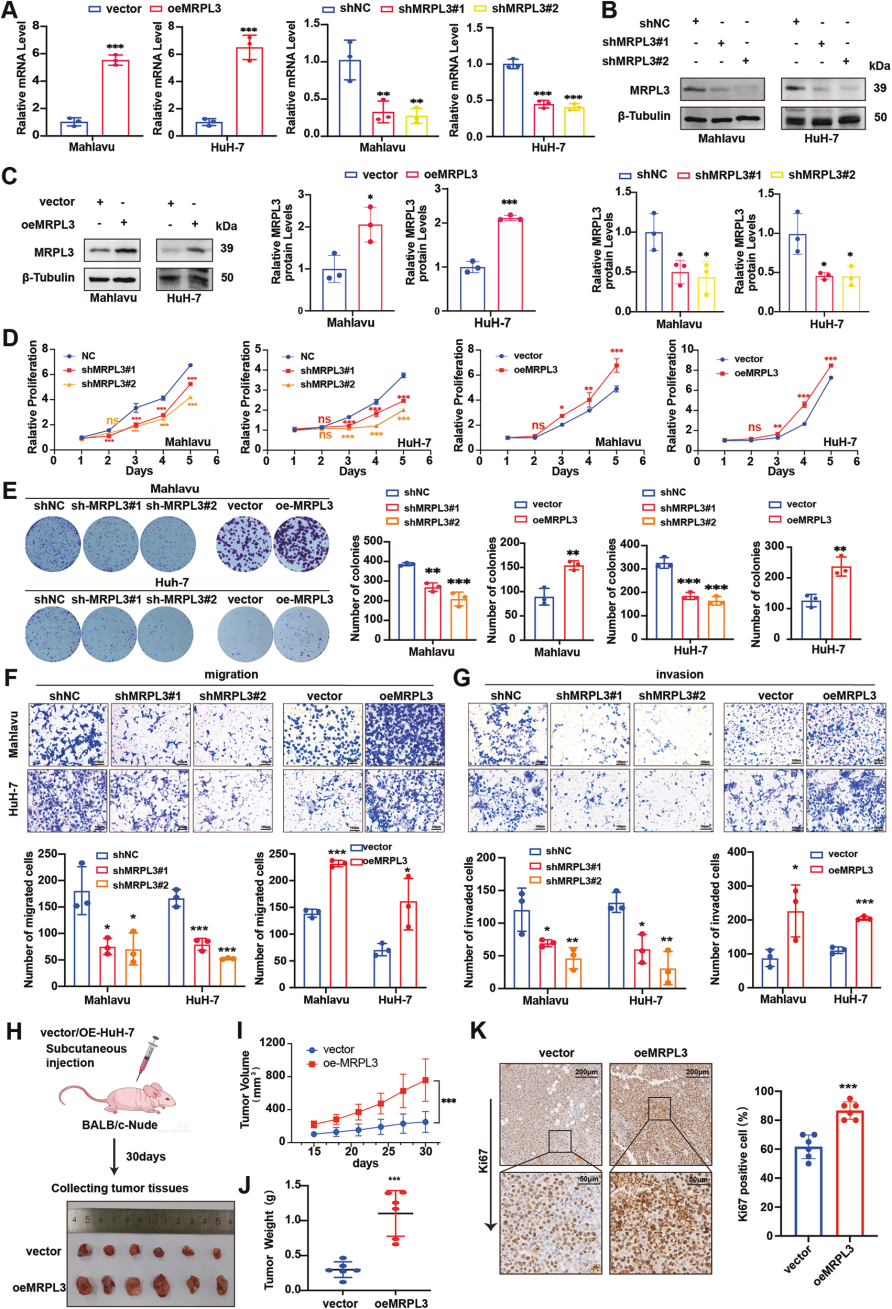
MRPL3 promotes HCC cell proliferation, migration, and invasion. Validation of MRPL3 knockdown/overexpression in Mahlavu and HuH-7 cells by qRT-PCR (**A**) and western blotting (**B, C**). **D** CCK-8 assay for cell proliferation. **E** Representative images and quantification of colony formation. **F, G** Transwell assays for migration and invasion (scale bar: 100 μm). **H** Schematic of the subcutaneous xenograft model and representative tumors from each group (*n* = 6). **I** Tumor volumes measured every 3 days and plotted as growth curves; two-way ANOVA. **J** Comparison of endpoint tumor weights; paired t-test. **K** Representative IHC staining of Ki-67 (scale bar: 50 μm) with quantification; unpaired t-test. Data are presented as the mean ± SD (*n* ≥ 3). Significance: ns, not significant; *P < 0.05; **P < 0.01; ***P < 0.001. HCC hepatocellular carcinoma, qRT-PCR quantitative reverse transcription polymerase chain reaction, SD standard deviation.

To define the functional role of MRPL3 in HCC progression, we first compared its basal expression across cell lines. MRPL3 expression was significantly higher in HCC cells than in immortalized hepatocytes (THLE-2) (Supplementary Fig. 2A, B). We subsequently modulated MRPL3 expression in Mahlavu and HuH-7 cells by knockdown and overexpression, with efficient manipulation confirmed (Fig. 3A–C). MRPL3 knockdown markedly decreased proliferative and clonogenic abilities in CCK-8 and colony-formation assays, whereas overexpression produced the opposite effect (Fig. 3D, E). Wound-healing and Transwell assays further demonstrated that MRPL3 depletion suppressed cell migration and invasion, whereas overexpression enhanced these processes (Supplementary Figs. 2C and 3F, G).

A subcutaneous xenograft model was employed to evaluate tumor growth in vivo. Consistent with the in vitro findings, tumors in the oeMRPL3 group were visibly larger (Fig. 3H), with significantly increased endpoint volume and weight compared with controls (Fig. 3I, J). IHC also revealed elevated Ki-67 staining in oeMRPL3 tumors (Fig. 3K). Consistently, in a complementary xenograft model using MRPL3-knockdown cells, tumor growth was markedly suppressed, as reflected by reduced tumor volume and weight, accompanied by decreased Ki-67 staining in tumor tissues (Supplementary Fig. 2D-G). Collectively, these findings demonstrate that MRPL3 significantly enhances HCC cell proliferation, migration, and invasion in vitro and facilitates tumor growth in vivo, indicating its pro-tumorigenic role in HCC progression and metastasis.

### MRPL3 improves mitochondrial function in HCC cells

**Fig. 4:**
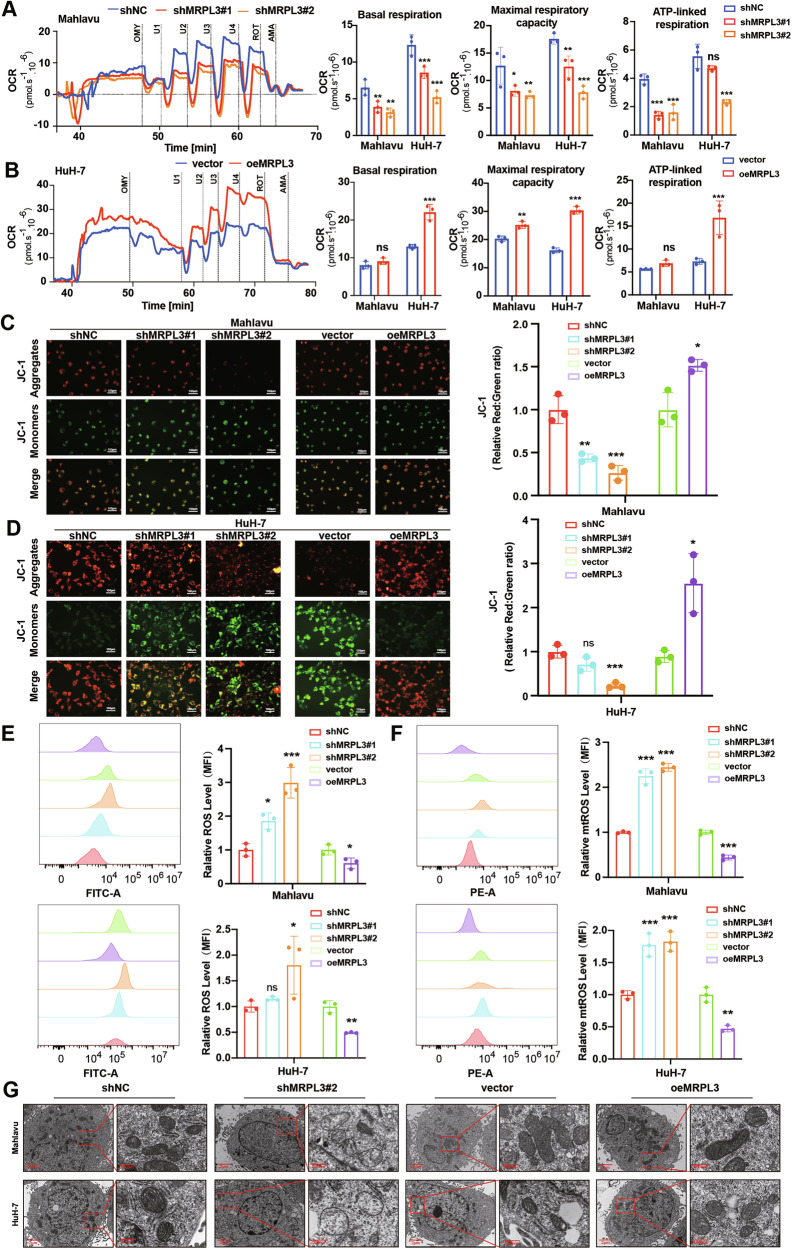
MRPL3 improves mitochondrial function in HCC cells. High-resolution respirometry with the Oroboros O2k in Mahlavu (**A**) and HuH-7 (**B**) cells. Representative oxygen consumption rate (OCR) traces are shown under basal conditions and following sequential additions of oligomycin, the uncoupler FCCP, and rotenone + antimycin A, with quantification of basal respiration, maximal respiration, and ATP-linked respiration. JC-1 staining to assess changes in mitochondrial membrane potential (ΔΨm) after MRPL3 knockdown or overexpression in Mahlavu (**C**) and HuH-7 (**D**) cells (scale bar: 100 μm). **E** Flow cytometry (FACS) showing representative histograms and mean fluorescence intensity (MFI) quantification of cellular reactive oxygen species (ROS). **F** FACS analysis of mitochondrial superoxide using MitoSOX, with representative histograms and MFI quantification. **G** Transmission electron microscopy (TEM) images of mitochondrial ultrastructure (scale bar: 2 μm; inset: 0.5 μm). Data are presented as the mean ± SD (*n* ≥ 3). Significance: ns, not significant; *P < 0.05; **P < 0.01; ***P < 0.001. HCC hepatocellular carcinoma, FCCP carbonyl cyanide-4-(trifluoromethoxy)phenylhydrazone, SD standard deviation.

To determine the impact of MRPL3 on mitochondrial function, high-resolution respirometry (Oroboros O2k) was performed in HCC cells with MRPL3 knockdown or overexpression. MRPL3 depletion significantly decreased basal, maximal, and ATP-linked respiration and spare respiratory capacity, whereas overexpression produced the opposite effect (Fig. 4A, B and Supplementary Fig. 3A–D). JC-1 staining indicated that MRPL3 knockdown decreased mitochondrial membrane potential (ΔΨm), whereas overexpression increased it (Fig. 4C, D). Flow cytometry revealed elevated cellular and mitochondrial ROS after MRPL3 knockdown, both of which were reduced by MRPL3 overexpression (Fig. 4E, F). TEM further demonstrated mitochondrial fragmentation and cristae swelling in MRPL3-depleted cells, whereas overexpression preserved mitochondrial ultrastructure relative to controls (Fig. 4G). ECAR analysis showed increased glycolysis and glycolytic capacity after MRPL3 knockdown, while MRPL3 overexpression had minimal effects (Supplementary Fig. 3E, F). In addition, MRPL3 depletion reduced the levels of the mtDNA-encoded respiratory chain subunits ND1 and COX I (Supplementary Fig. 3G, H).

**Fig. 5:**
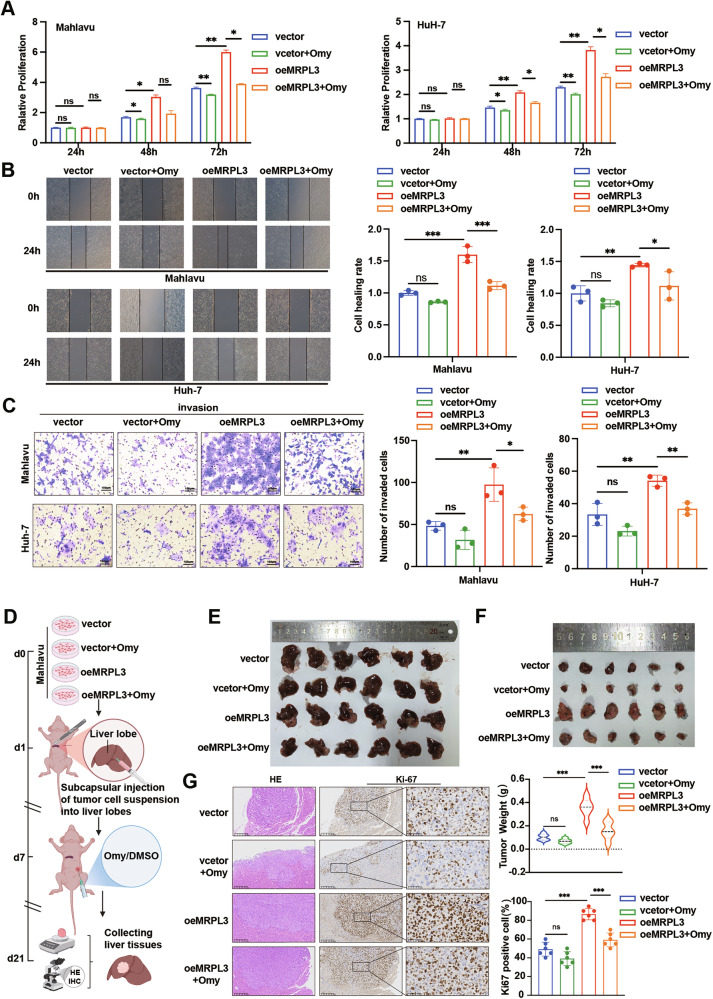
Oligomycin partially reverses MRPL3 overexpression-induced proliferation, migration, invasion, and tumor growth in HCC. **A** CCK-8 assays for cell proliferation in Mahlavu and HuH-7 cells treated with vector, vector + Omy, oeMRPL3, or oeMRPL3 + Omy. **B** Representative images and quantification of wound-healing assays. **C** Representative images and quantification of Transwell invasion assays. **D** Schematic of the orthotopic liver tumor model and treatment protocol. **E** Representative gross images of orthotopic liver tumors from each group. **F** Representative images and quantification of tumor weights in the indicated groups. **G** Representative H&E and Ki-67 staining of orthotopic tumor tissues, with quantification of Ki-67-positive cells. Data are presented as the mean ± SD (*n* ≥ 3). Significance: ns, not significant; *P < 0.05; **P < 0.01; ***P < 0.001. HCC hepatocellular carcinoma, H&E hematoxylin and eosin, SD standard deviation.

To evaluate whether mitochondrial respiration is required for MRPL3-driven malignant phenotypes, MRPL3-overexpressing cells were treated with the OXPHOS inhibitor oligomycin. Oligomycin markedly attenuated MRPL3-induced increases in cell proliferation, migration, and invasion (Fig. 5A–C), indicating that the tumor-promoting effects of MRPL3 depend on oxidative phosphorylation. Consistently, oligomycin treatment significantly suppressed tumor growth in an orthotopic liver tumor model (Fig. 5D–G), further supporting a critical role for OXPHOS in MRPL3-mediated tumor progression.

Collectively, these results indicate that MRPL3 supports mitochondrial integrity and bioenergetic function in HCC cells.

### MRPL3 influences mitochondrial function in HCC cells through the PGC-1α/TFAM signaling pathway

**Fig. 6:**
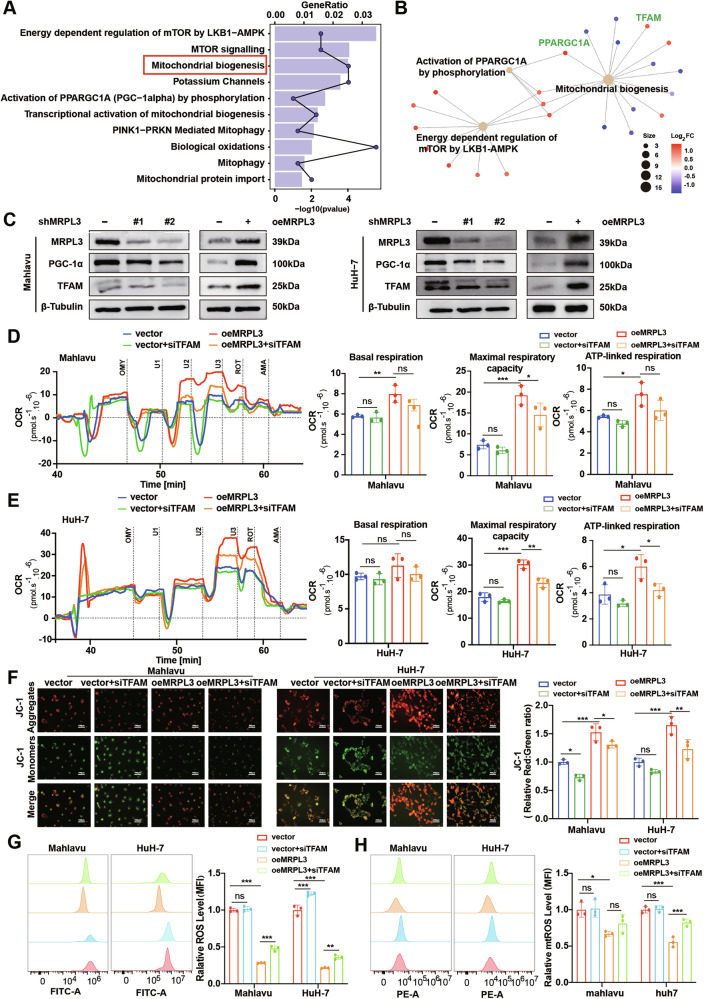
MRPL3 regulates mitochondrial function via the PGC-1α/TFAM pathway in HCC cells. Reactome pathway enrichment based on RNA-seq from MRPL3-overexpressing (oeMRPL3) versus vector control cells (**A**), and a network map linking selected pathways with associated genes (**B**). **C** Western blot analysis showing PGC-1α and TFAM protein levels after MRPL3 knockdown or overexpression. High-resolution respirometry (Oroboros O2k) in Mahlavu (**D**) and HuH-7 (**E**) cells with MRPL3 overexpression combined with TFAM knockdown; representative oxygen consumption rate (OCR) traces and quantification of basal respiration, maximal respiration, and ATP-linked respiration are shown. **F** JC-1 staining to assess changes in mitochondrial membrane potential (ΔΨm) following MRPL3 overexpression with TFAM knockdown (scale bar: 100 μm). **G** Flow cytometry (FACS) showing representative histograms and mean fluorescence intensity (MFI) quantification of cellular reactive oxygen species (ROS) under the same conditions. **H** FACS analysis of mitochondrial superoxide (MitoSOX) with representative histograms and MFI quantification. Data are presented as the mean ± SD (*n* ≥ 3). Significance: ns, not significant; *P < 0.05; **P < 0.01; ***P < 0.001. HCC hepatocellular carcinoma, SD standard deviation.

To elucidate how MRPL3 regulates mitochondrial function, we performed RNA-seq on HuH-7 cells overexpressing MRPL3 (oeMRPL3) versus vector controls. Reactome enrichment analysis revealed significant enrichment of AMPK signaling and mitochondrial biogenesis pathways (Fig. 6A). Considering the pivotal function of AMPK in metabolic regulation and mitochondrial formation [28, 29], further analysis of the dataset identified PGC-1α as a key regulatory node (Fig. 6B). PGC-1α promotes mitochondrial biogenesis by enhancing NRF1/NRF2 expression, which subsequently activates TFAM [30, 31].

Consistent with this model, PGC-1α and TFAM expression positively correlated with MRPL3 levels in HCC cells (Fig. 6C and Supplementary Fig. 4A, B). Notably, siRNA-mediated TFAM knockdown abrogated the MRPL3-induced improvements in mitochondrial function, reversing increases in oxygen consumption rate (Fig. 6D, E and Supplementary Fig. 4C, D) and mitochondrial membrane potential (Fig. 6F) and negating reductions in cellular and mitochondrial ROS (Fig. 6G, H).

Together, these findings identify the PGC-1α/TFAM axis as an essential mediator of MRPL3-driven mitochondrial remodeling in HCC.

### MRPL3 modulates the PGC-1α/TFAM signaling pathway through association with TOMM40

**Fig. 7:**
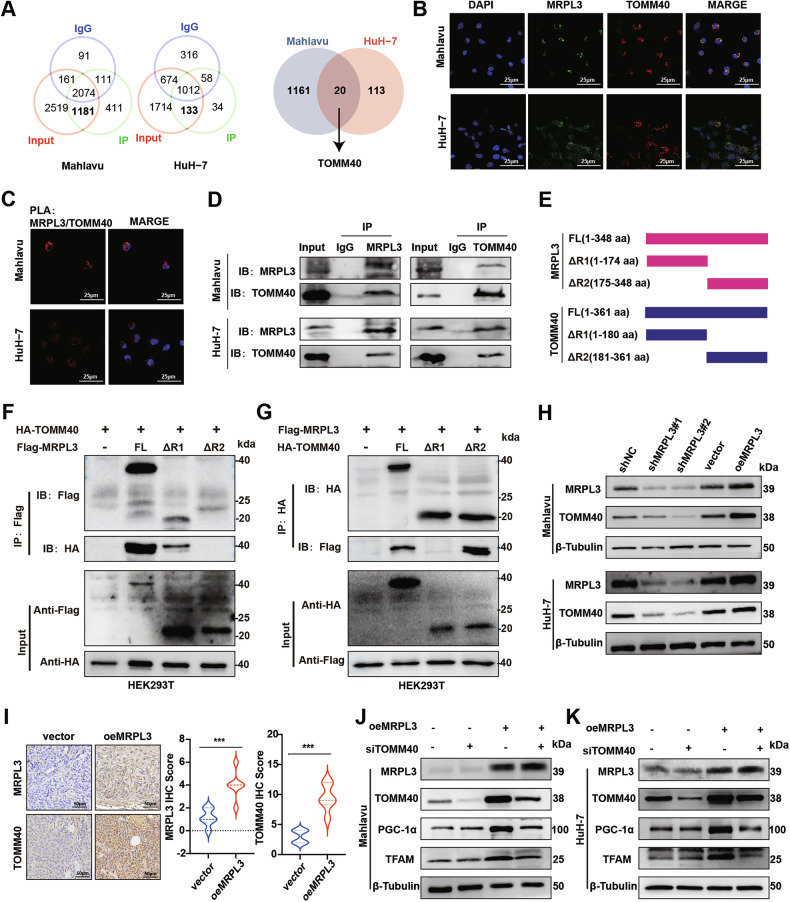
MRPL3 regulates the PGC-1α/TFAM pathway through interaction with TOMM40. **A** Venn diagram of candidate interactors identified by proteomic screening in Mahlavu and HuH-7 cells. **B** Immunofluorescence (IF) showing colocalization of MRPL3 (green) and TOMM40 (red) with DAPI (blue) nuclear staining in HCC cells (scale bar: 25 μm). **C** Proximity ligation assay (PLA) depicting representative MRPL3–TOMM40 interaction signals in Mahlavu and HuH-7 cells (scale bar: 25 μm). **D** Co-immunoprecipitation (Co-IP) validating the interaction between MRPL3 and TOMM40 in HCC cells. **E** Schematic of Flag-tagged MRPL3 truncation mutants and HA-tagged TOMM40 truncation mutants. HEK293T cells co-transfected with Flag-MRPL3 plus HA-tagged TOMM40 truncations (**F**), or HA-TOMM40 plus Flag-tagged MRPL3 truncations (**G**); cell lysates were subjected to immunoprecipitation (IP) followed by immunoblotting (IB) with anti-Flag and anti-HA. FL, full length. **H** Western blot analysis of TOMM40 protein levels in HCC cells upon MRPL3 knockdown or overexpression. **I** Representative immunohistochemistry (IHC) of MRPL3 and TOMM40 in xenograft tumors with quantification. Western blot analysis of PGC-1α (**J**) and TFAM (**K**) protein levels in HCC cells overexpressing MRPL3 with concurrent TOMM40 knockdown. HCC, hepatocellular carcinoma.

To determine how MRPL3 modulates the PGC-1α/TFAM axis, MRPL3-interacting proteins were immunoprecipitated and analyzed by LC–MS/MS in Mahlavu and HuH-7 cells. Twenty candidate interactors were identified, among which TOMM40, a mitochondrial outer membrane protein, was of particular interest (Fig. 7A and Supplementary Fig. 5A). TOMM40, a core subunit of the translocase of the outer membrane (TOM) complex, is crucial for the import of nuclear-encoded proteins into mitochondria [32, 33] and is frequently upregulated in cancers, where it correlates with poorprognosis [34–36].

IF, PLA, and Co-IP demonstrated an association between MRPL3 and TOMM40 in HCC cells (Fig. 7B–D). To define the interaction interface, a panel of Flag-tagged MRPL3 truncations and HA-tagged TOMM40 truncations was constructed (Fig. 7E). Domain mapping revealed that the ΔR1 region of MRPL3 (1–174 aa) and the ΔR2 region of TOMM40 (181–361 aa) are required for their association (Fig. 7F, G). To determine whether MRPL3 interacts with TOMM40 within mitochondria, purified mitochondrial fractions were isolated from HCC cells. Western blotting confirmed successful mitochondrial enrichment and detected both MRPL3 and TOMM40 in the mitochondrial fraction (Supplementary Fig. 5B). Further Co-IP of isolated mitochondrial proteins showed that MRPL3 remained associated with TOMM40 within mitochondria (Supplementary Fig. 5C). Functionally, MRPL3 levels markedly influenced TOMM40 protein abundance (Fig. 7H, I and Supplementary Fig. 6A). qRT-PCR showed that MRPL3 modulation did not alter TOMM40 mRNA levels, indicating that TOMM40 is not regulated at the transcriptional level (Supplementary Fig. 5D, E). By contrast, cycloheximide chase assays demonstrated a prolonged TOMM40 half-life upon MRPL3 overexpression, and MG-132 treatment increased TOMM40 abundance while attenuating the vector–oeMRPL3 differences (Supplementary Fig. 5F–I). Together, these results show that MRPL3 increases TOMM40 abundance through protein stabilization, not transcriptional regulation. Moreover, TOMM40 knockdown suppressed PGC-1α and TFAM protein expression in HCC cells (Fig. 7J, K and Supplementary Fig. 6B, C). These findings suggest that MRPL3 modulates the PGC-1α/TFAM signaling pathway through its association with TOMM40.

### MRPL3 regulates mitochondrial function via TOMM40 to promote HCC cell proliferation, migration, and invasion

**Fig. 8:**
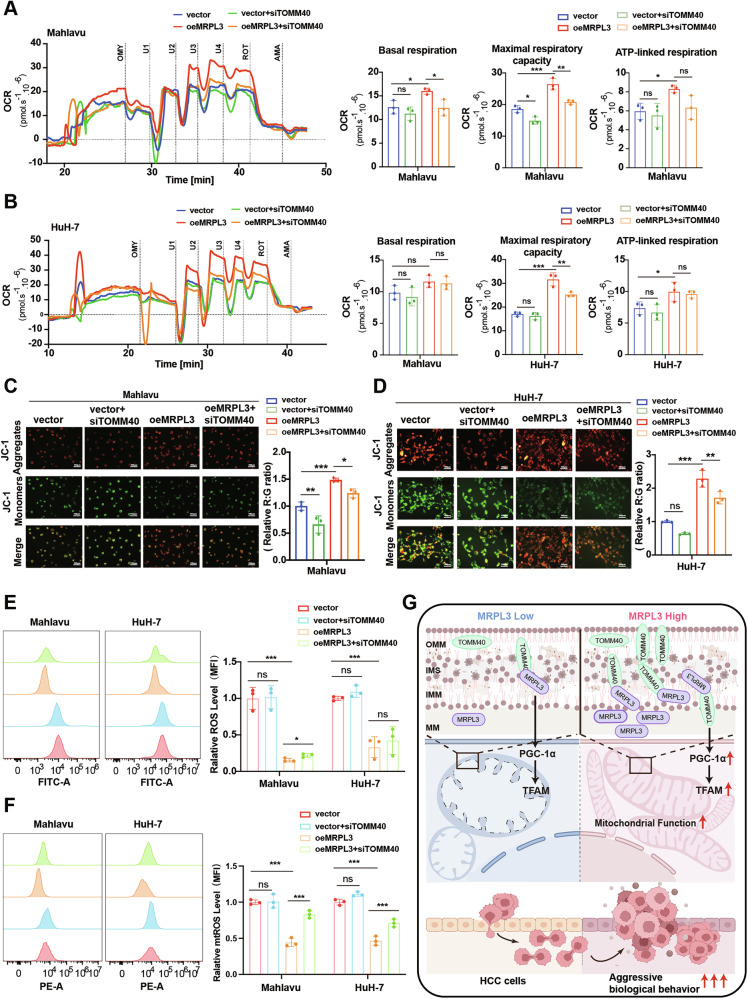
MRPL3 regulates mitochondrial function in HCC cells via interaction with TOMM40. High-resolution respirometry (Oroboros O2k) in Mahlavu (**A**) and HuH-7 (**B**) cells overexpressing MRPL3 with concurrent TOMM40 knockdown; representative oxygen consumption rate (OCR) traces and quantification of basal respiration, maximal respiration, and ATP-linked respiration are shown. JC-1 staining to assess changes in mitochondrial membrane potential (ΔΨm) under the same conditions in Mahlavu (**C**) and HuH-7 (**D**) cells (scale bar: 100 μm). **E** Flow cytometry (FACS) showing representative histograms and mean fluorescence intensity (MFI) quantification of cellular reactive oxygen species (ROS). **F** FACS analysis of mitochondrial superoxide (MitoSOX) with representative histograms and MFI quantification. **G** Schematic illustrating how MRPL3 modulates mitochondrial function via the TOMM40/PGC-1α/TFAM axis in early-recurrent HCC. Data are presented as the mean ± SD (*n* ≥ 3). Significance: ns, not significant; *P < 0.05; **P < 0.01; ***P < 0.001. HCC hepatocellular carcinoma, SD standard deviation.

To determine whether TOMM40 mediates the effects of MRPL3 on mitochondrial function, TOMM40 was silenced in MRPL3-overexpressing HCC cells. High-resolution respirometry demonstrated that TOMM40 knockdown counteracted the MRPL3-induced improvements in mitochondrial respiration, encompassing basal, maximal, and ATP-linked respiration and spare respiratory capacity (Fig. 8A, B and Supplementary Fig. 7A, B). JC-1 staining showed that TOMM40 depletion attenuated the MRPL3-driven increase in mitochondrial membrane potential (Fig. 8C, D). Flow cytometry further demonstrated that TOMM40 knockdown reversed the MRPL3-mediated reduction of cellular and mitochondrial ROS (Fig. 8E, F).

Functionally, CCK-8, colony-formation, wound-healing, and Transwell assays demonstrated that silencing TOMM40 significantly inhibited the MRPL3-induced enhancements in cell proliferation, migration, and invasion (Supplementary Fig. 7C–G). Together, these findings establish TOMM40 as an essential mediator through which MRPL3 activates the PGC-1α/TFAM axis to enhance mitochondrial function and drive malignant phenotypes in HCC cells.

## Discussion

Despite advances in surveillance and therapeutic strategies that have modestly improved OS, clinical outcomes for HCC remain unsatisfactory owing to the high rate of early recurrence after resection [37, 38]. Early recurrence, often driven by occult micrometastases, substantially compromises survival and quality of life. To elucidate its molecular underpinnings, we performed proteomic profiling to identify key regulators and pathways underlying early recurrence. Metabolic reprogramming is crucial in HCC progression, making tumor metabolism a promising therapeutic target. Here, we demonstrate that MRPL3 augments OXPHOS and improves mitochondrial homeostasis in HCC cells. Mechanistically, as depicted in Fig. 8G, MRPL3 associates with TOMM40 to form a protein complex that contributes to activation of the PGC-1α/TFAM axis, enhancing mitochondrial function and promoting malignant phenotypes such as proliferation, migration, and invasion. These findings uncover a previously unrecognized mechanism by which MRPL3 drives early recurrence of HCC and establish MRPL3 as a critical regulator of mitochondrial homeostasis and a potential therapeutic target.

Mitoribosomal proteins are essential for preserving mitochondrial structural and functional integrity and play important roles in tumorigenesis and progression [39]. Over 80 mitoribosomal proteins have been identified in mammals, with several family members implicated in cancer [25, 40, 41]. MRPL3, a nuclear-encoded component of the mitochondrial large ribosomal subunit, is imported into mitochondria after cytosolic translation and participates in mitoribosome assembly and functional maintenance [21]. Although MRPL3 has been proposed as a prognostic marker in HCC [23, 42], its specific role and mechanisms in disease progression remain poorly understood. In this study, we observed markedly elevated MRPL3 expression in human HCC tissues, with higher levels associated with MVI and early postoperative recurrence (Supplementary Table 1 and Fig. 2B–H). As a hallmark of aggressive tumor behavior, MVI is closely associated with postoperative recurrence and poor surgical outcomes. High MRPL3 expression consistently correlated with poorer DFS and OS, and multivariate Cox analyses identified it as an independent adverse prognostic factor for both endpoints (Fig. 2I, J and Supplementary Tables 4 and 5). Although the DFS difference did not reach statistical significance in Kaplan–Meier analysis, the overall trend together with multivariate modeling suggests a potential prognostic value of MRPL3 that warrants further validation in larger cohorts. Postoperative prognostic nomograms integrating MRPL3 expression and the AJCC stage showed good predictive performance and calibration for DFS and OS (Supplementary Fig. 1D–I). Subsequent in vitro and in vivo functional and mechanistic experiments provided comprehensive and compelling evidence for the oncogenic role of MRPL3.

The Warburg effect is widely recognized as a dominant metabolic feature of many cancer cells and reportedly promotes HCC progression [43–45]. However, mounting evidence indicates that certain tumors rely heavily on mitochondrial function to sustain growth and dissemination [46–49]. Given the metabolic heterogeneity of HCC, the tumor-promoting role of mitochondrial metabolism warrants careful consideration [50, 51]. For example, SIRT2-mediated P5CS deacetylation enhances mitochondrial respiration and tumor growth in HCC [52]; SNX17 activates the STAT3/c-Myc pathway via a reverse-transcriptase–dependent mechanism to augment OXPHOS and mitochondrial biogenesis, thereby driving HCC progression [53]; moreover, Li et al. reported that WNK1 interacts with KEAP1 to stabilize NRF2, strengthening cellular defense against oxidative stress [54]. In this study, we demonstrated that MRPL3 improves mitochondrial function in HCC cells, as evidenced by increased OXPHOS, elevated mitochondrial membrane potential (ΔΨm), and reduced cellular and mitochondrial ROS. Consistent with these findings, ECAR profiling revealed only modest alterations in glycolytic parameters following MRPL3 manipulation, suggesting that the metabolic effects of MRPL3 are primarily associated with mitochondrial respiration, while glycolytic activity may undergo limited or compensatory adjustments. We have not yet examined whether MRPL3 modulates mitochondrial dynamics (such as fusion/fission or mitophagy), an interesting avenue for future studies. Accordingly, we cannot exclude the possibility that MRPL3 regulates mitochondrial function through additional mechanisms. Importantly, pharmacological inhibition of OXPHOS with oligomycin markedly attenuated MRPL3-induced malignant phenotypes both in vitro and in vivo. These findings provide direct evidence that MRPL3-driven metabolic reprogramming contributes to tumor progression and further suggest that a subset of HCC may preferentially depend on mitochondrial bioenergetics, rather than glycolysis, to support growth and dissemination.

As a central determinant of cellular metabolic regulation, mitochondrial biogenesis is essential for maintaining mitochondrial function and is tightly linked to tumor development and progression [55, 56]. In HCC, accumulating evidence implicates PGC-1α in tumor growth, metastasis, and therapeutic response [57–59]. PGC-1α acts as a key regulator of mitochondrial biogenesis by upregulating TFAM, which in turn boosts mitochondrial DNA replication and transcription, maintaining mitochondrial structural and functional balance—an essential process in both healthy and cancerous cells [60, 61]. The PGC-1α/TFAM pathway is primarily activated by metabolic sensors such as AMPK and SIRT1 and is further modulated by CREB, AKT, and HIF signaling [55, 62, 63]. This study demonstrates that MRPL3 promotes activation of the PGC-1α/TFAM axis. Consistent with the mitoribosomal function of MRPL3, MRPL3 depletion reduced the protein levels of the mtDNA-encoded respiratory chain subunits ND1 and COX I, suggesting that MRPL3 promotes mitochondrial respiratory capacity, at least in part, by maintaining the abundance of mtDNA-encoded OXPHOS components.

Mechanistically, we focused on TOMM40, the pore-forming core subunit of the TOM complex, which mediates the import of most nuclear-encoded mitochondrial proteins. Beyond protein import, TOMM40 participates in autophagy and mitochondrial homeostasis through protein–protein interactions, linking it to cancer, neurodegeneration, innate immunity, and aging [64–67]. Our truncation mapping indicates a specific association between MRPL3 ΔR1 (1–174 aa) and TOMM40 ΔR2 (181–361 aa). Notably, these regions do not correspond to annotated functional domains in MRPL3 or TOMM40, indicating that our truncation analysis identifies minimal interacting regions rather than defined structural domains. Given that MRPL3 is localized in the mitochondrial matrix, whereas TOMM40 resides in the outer mitochondrial membrane, a direct transmembrane interaction would be topologically challenging. Mitochondrial purification and mitochondrial co-immunoprecipitation further support that this association is maintained within isolated mitochondrial fractions. Together with TEM evidence showing cristae disruption and inner-membrane perturbation upon MRPL3 depletion, we hypothesize that membrane disorganization or contact-site microdomains in HCC may bring matrix-localized MRPL3 into close proximity with outer-membrane TOMM40. This may facilitate the formation of a mitochondrial protein complex that contributes to activation of the PGC-1α/TFAM axis and improved mitochondrial function. The precise molecular interface and transmembrane communication require further definition. Elucidating this pathway will advance our understanding of tumor subsets that depend on mitochondrial OXPHOS rather than the canonical Warburg program to sustain growth.

A limitation of this study is the absence of a resection-based orthotopic recurrence model, and future studies incorporating such models will be needed to more faithfully recapitulate postoperative relapse after curative surgery.

In summary, our study identifies MRPL3 as a promoter of HCC malignancy and early recurrence. Mechanistically, MRPL3 associates with TOMM40 and enhances activation of the PGC-1α/TFAM axis, thereby improving mitochondrial function and promoting proliferation, migration, and invasion. These findings highlight a role for MRPL3-driven mitochondrial metabolic reprogramming in HCC progression and suggest that targeting this pathway may represent a potential therapeutic strategy for HCC.

## Supplementary information

Supplementary Figure 1

Supplementary Figure 2

Supplementary Figure 3

Supplementary Figure 4

Supplementary Figure 5

Supplementary Figure 6

Supplementary Figure 7

The list of supplementary figure legends

Supplementary Table 1

Supplementary Table 2

Supplementary Table 3

Supplementary Table 4

Supplementary Table 5

Supplemental Methods

Original Western blots

## Data Availability

The datasets generated during and analyzed during the current study are available from the corresponding author on reasonable request.
