# Supplementary material for: MRPL3 enhances mitochondrial function via the TOMM40/PGC-1α/TFAM axis to drive early recurrence in hepatocellular carcinoma
Source: Cell Death Dis. 2026 Jun 9;17(1):721. doi: 10.1038/s41419-026-08968-8 (PMC13469937; doi:10.1038/s41419-026-08968-8)

F2-C

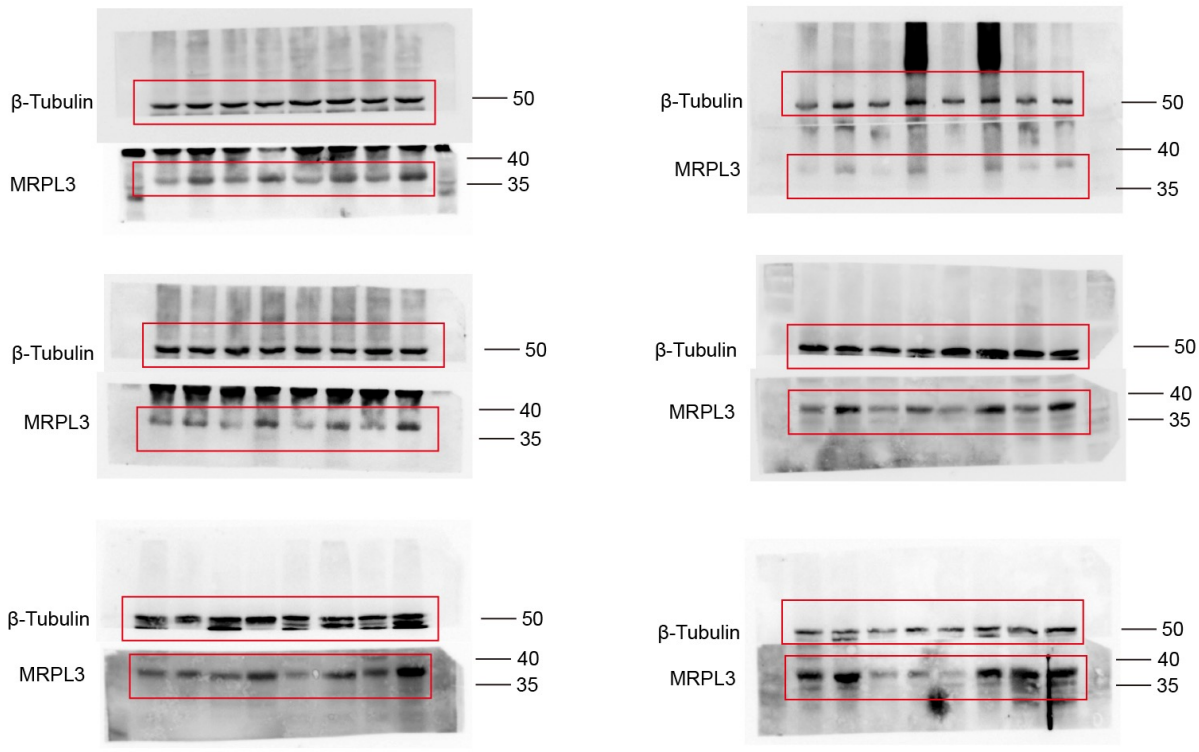

F3-B

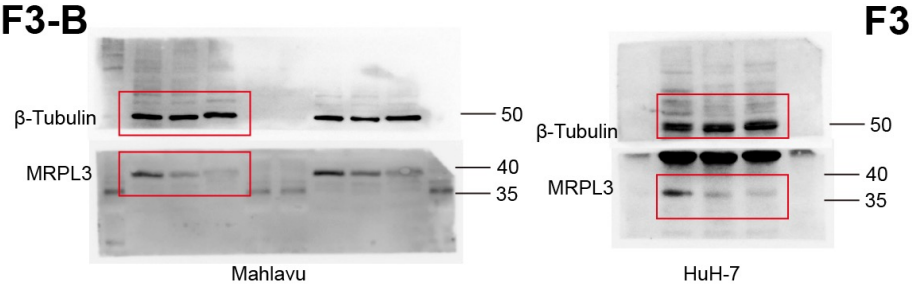

F3-C

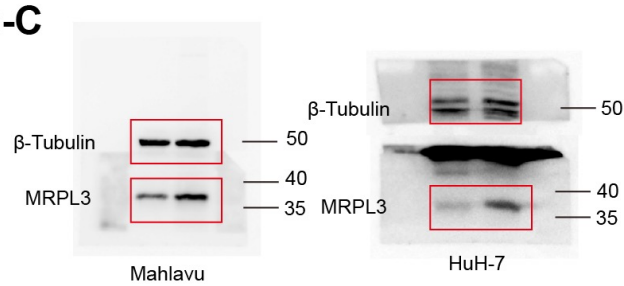

F6-C

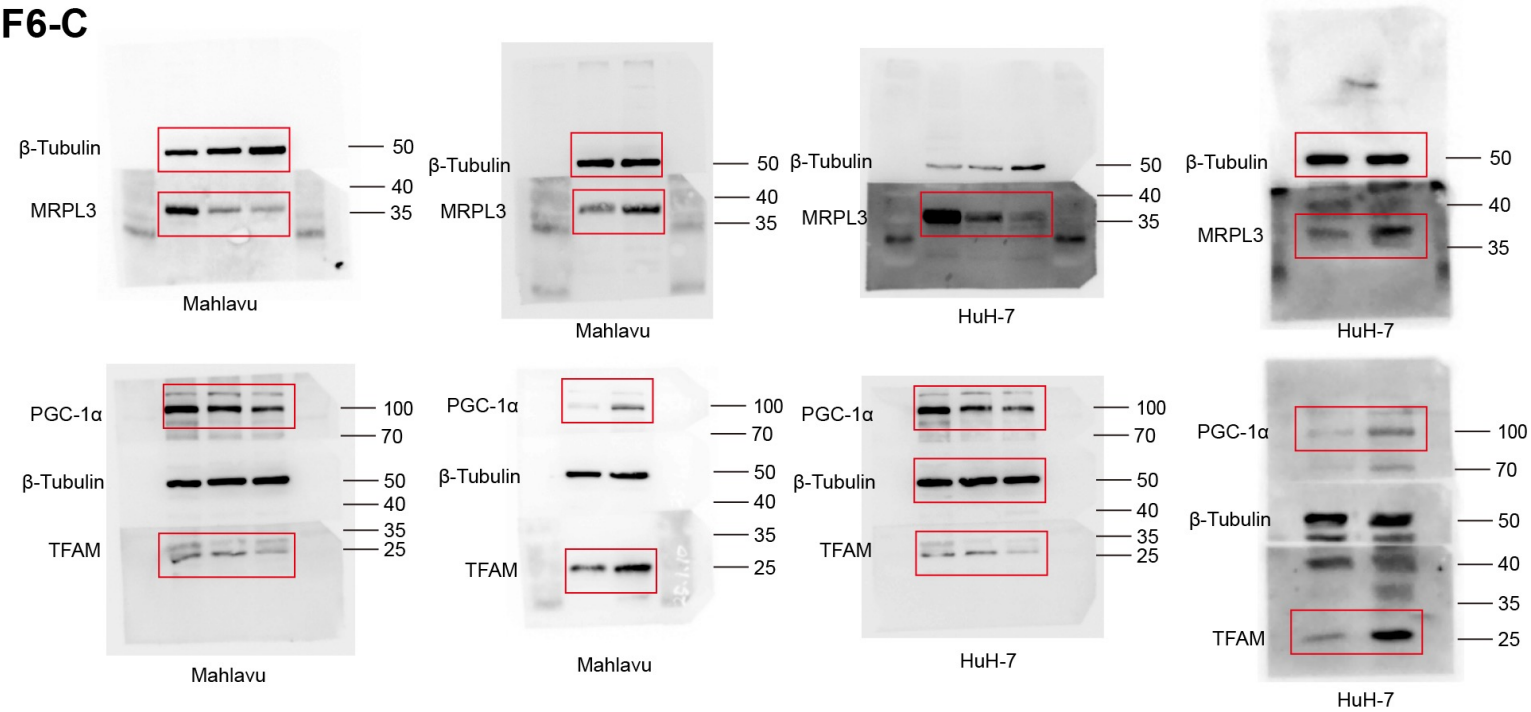

F7-D

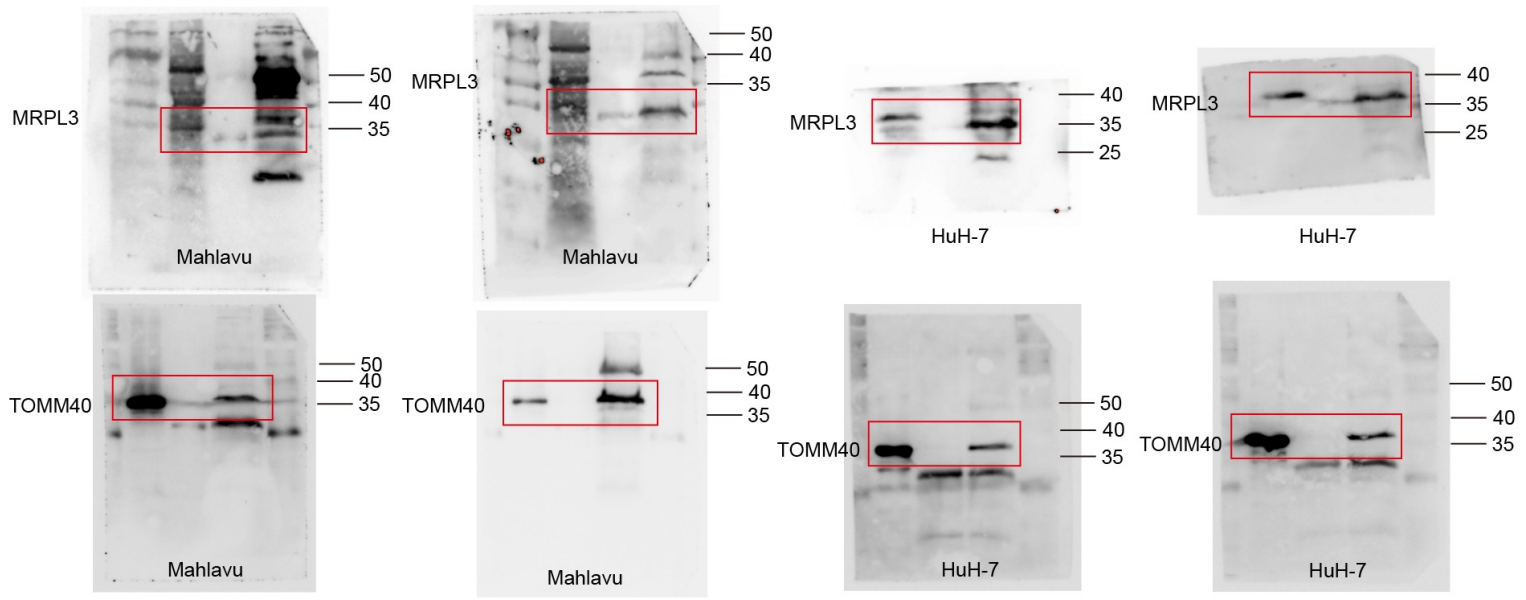

F7-F

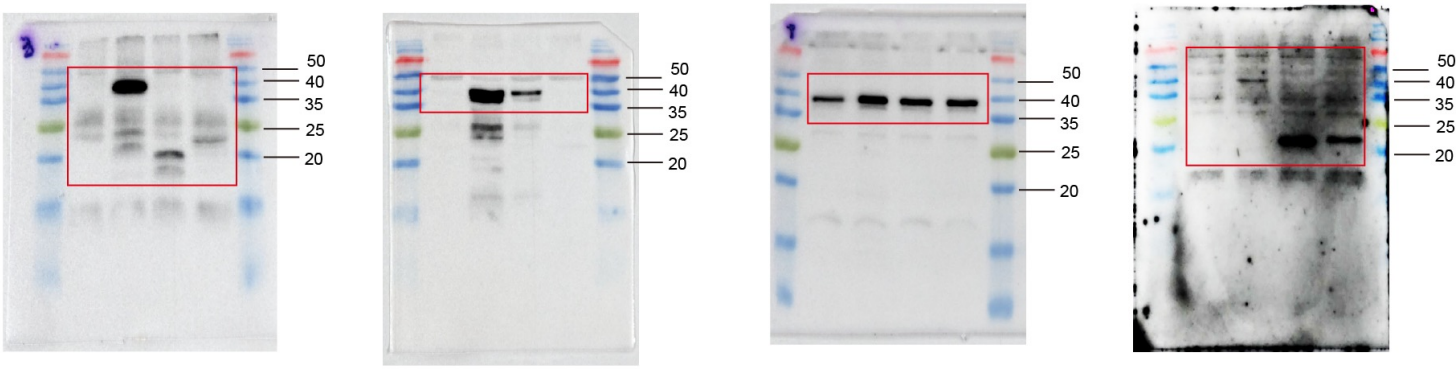

F7-G

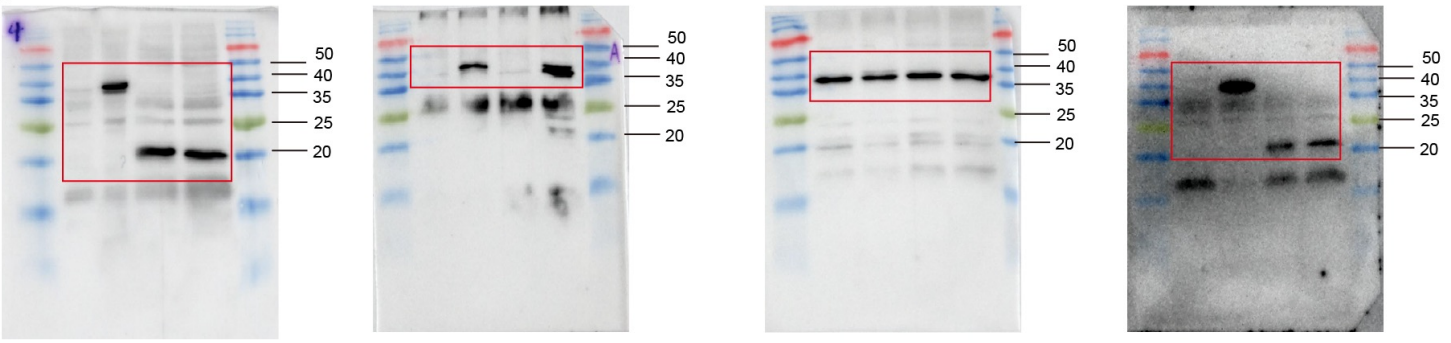

F7-H

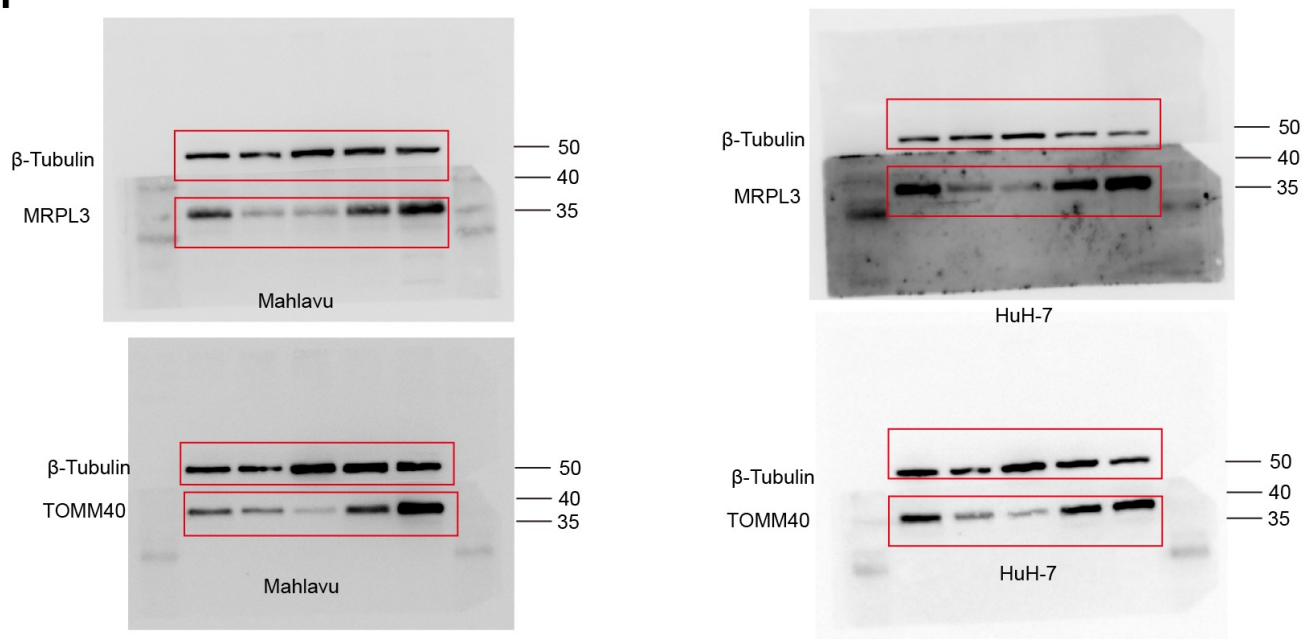

F7-J

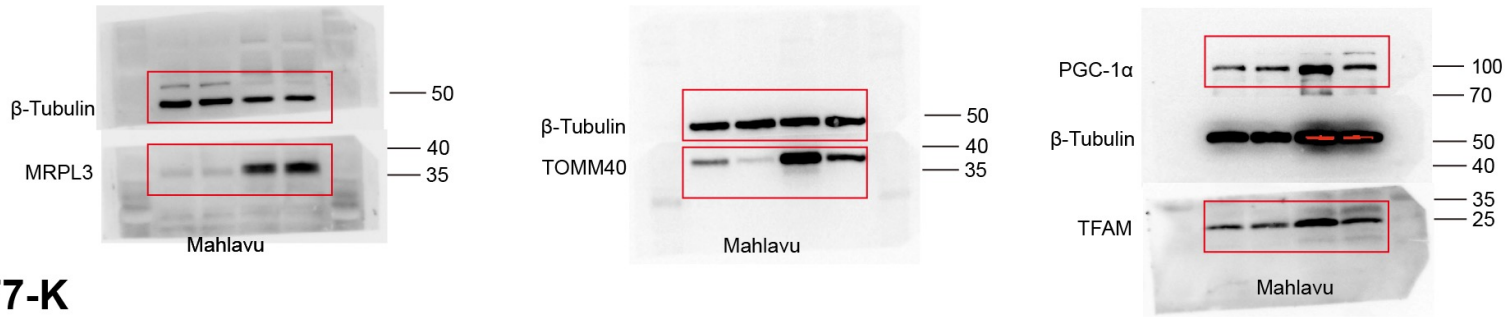

F7-K

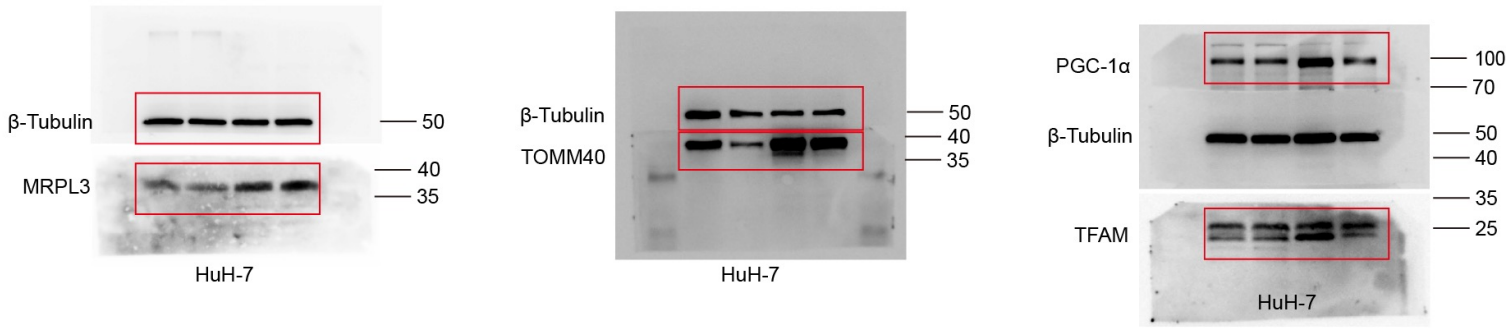

FS2-B

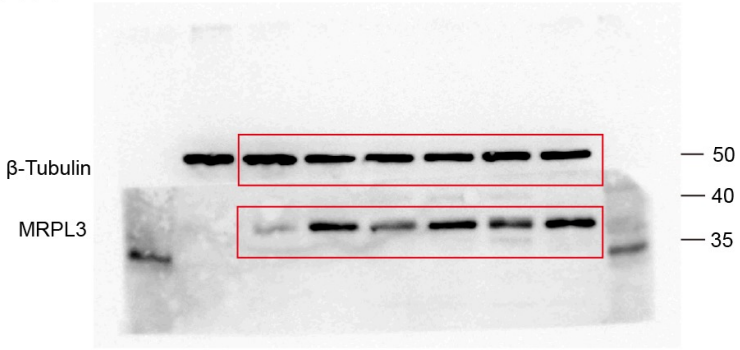

FS3-G

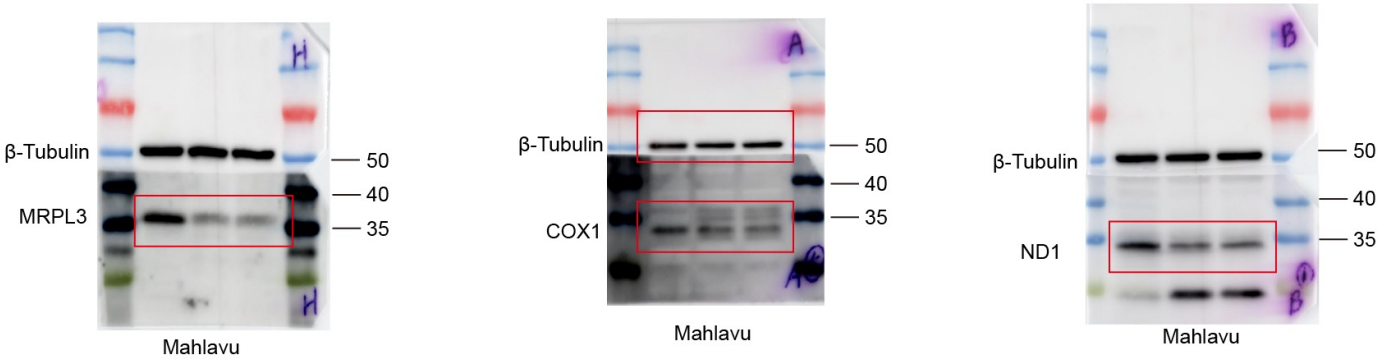

FS3-H

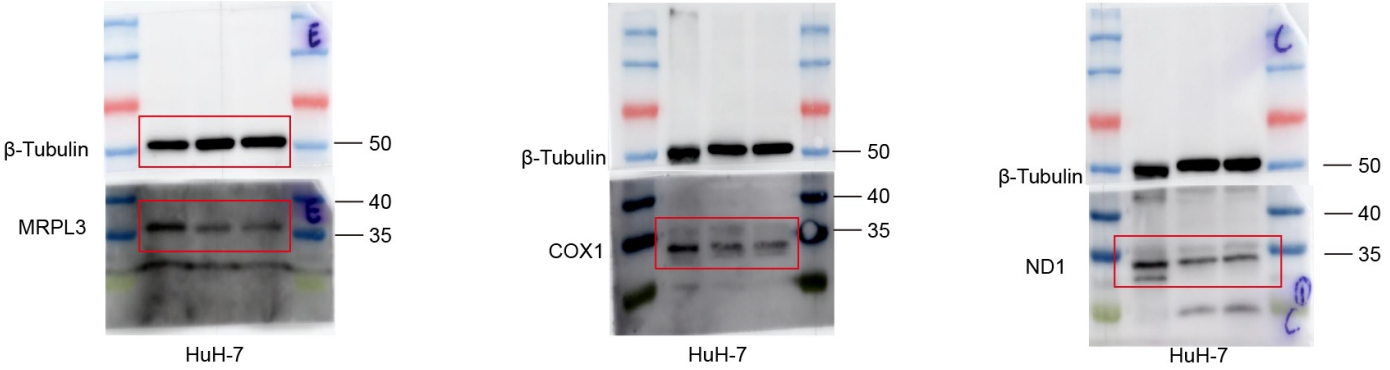

FS5-B

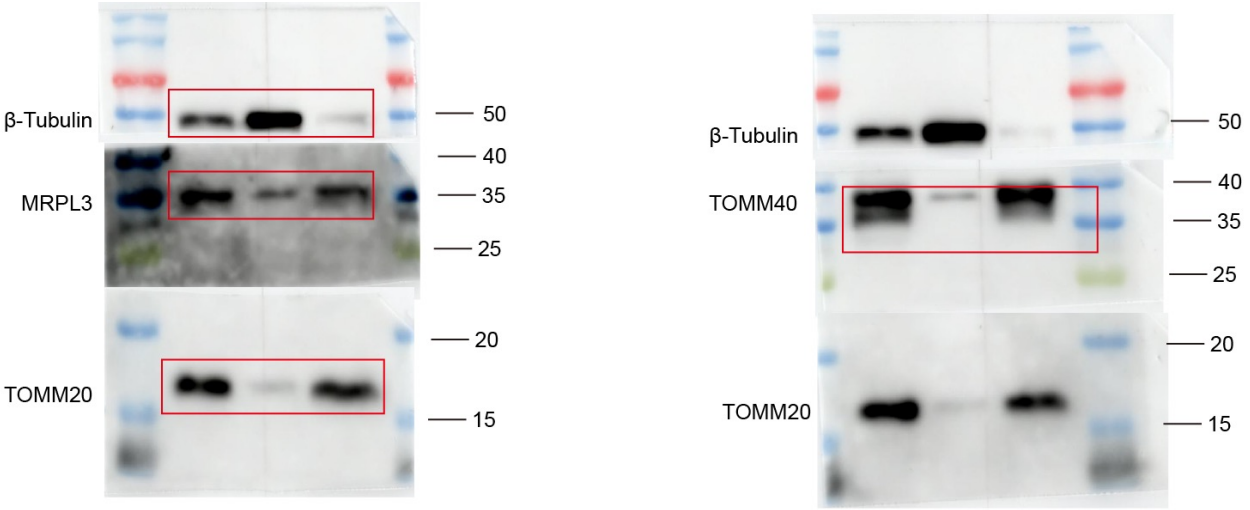

FS5-C

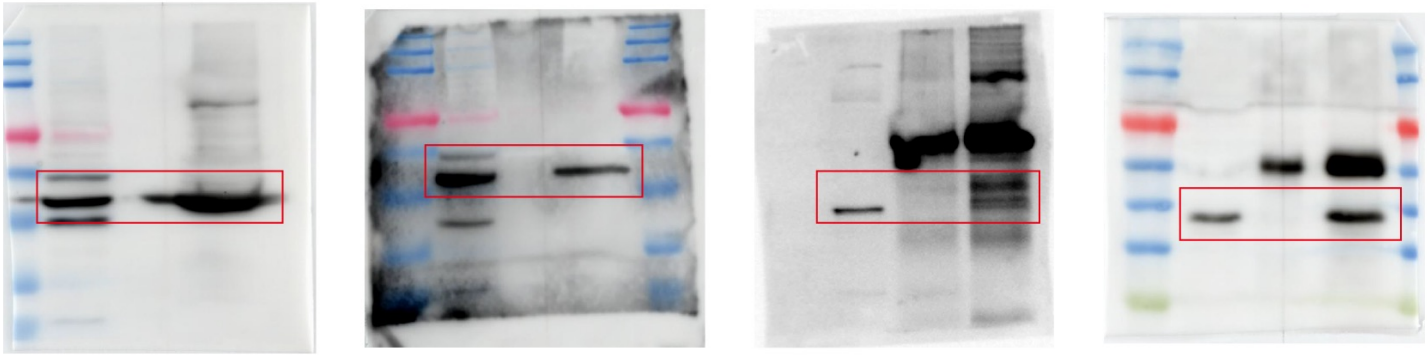

FS5-F

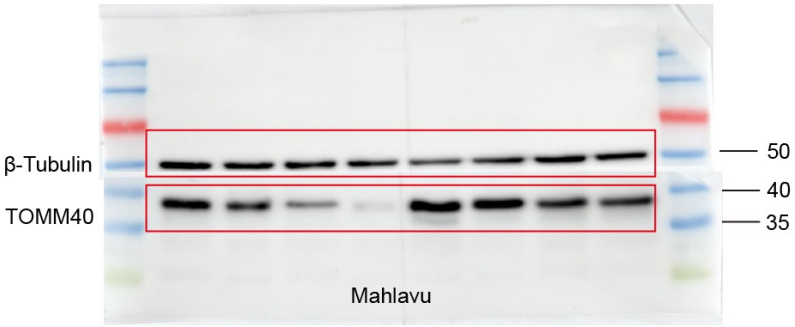

FS5-H

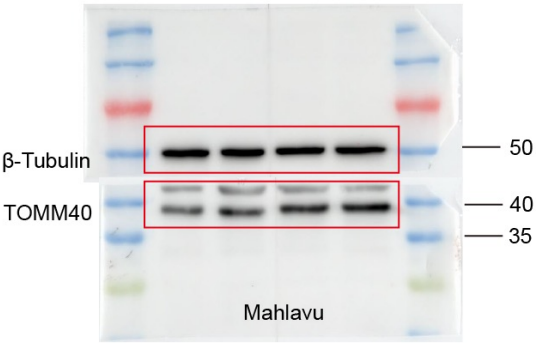

FS5-G

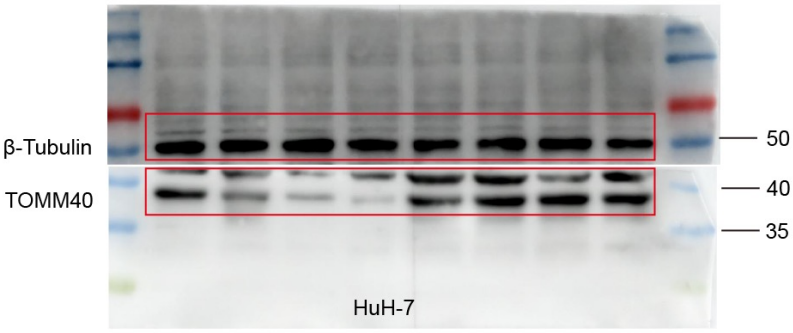

FS5-I

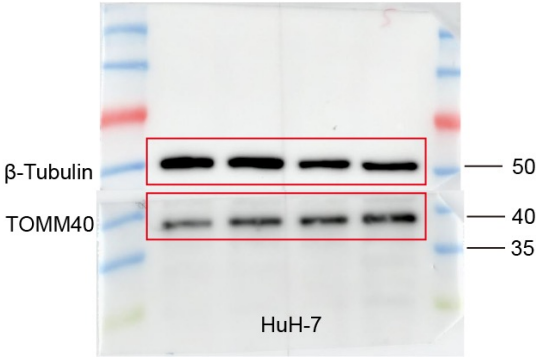

Supplement: Supplementary file 1 [file 41419_2026_8968_MOESM15_ESM.pdf]
