# Supplementary material for: MRPL3 enhances mitochondrial function via the TOMM40/PGC-1α/TFAM axis to drive early recurrence in hepatocellular carcinoma
Source: Cell Death Dis. 2026 Jun 9;17(1):721. doi: 10.1038/s41419-026-08968-8 (PMC13469937; doi:10.1038/s41419-026-08968-8)
Supplement: Supplementary file 2 [file 41419_2026_8968_MOESM10_ESM.docx]

**Table. S2 Reagent and Resource.**

| Reagent or Resource | Source | Identifier |
| --- | --- | --- |
| Antibodies |  |  |
| Anti-β-Tubulin antibody | Proteintech | Cat#10094-1-AP |
| Anti-MRPL3 antibody | Proteintech | Cat#16584-1-AP |
| Anti-MRPL3 antibody | SANTA CRUZ | Cat#sc-514217 |
| Anti-TOMM40 antibody | Proteintech | Cat#18409-1-AP |
| Anti-PGC-1α antibody | Abcam | Cat#ab191838 |
| Anti-TFAM antibody | Abcam | Cat#ab176558 |
| Anti-Ki67 antibody | Abcam | Cat#ab16667 |
| Anti-COX1 antibody | Beyotime | Cat#AG2674 |
| Anti-ND1 antibody | Beyotime | Cat#AF7515 |
| Goat Anti-Rabbit IgG antibody | Proteintech | Cat#66467-1-Ig |
| Goat Anti-Mouse IgG antibody | Abcam | Cat#ab190475 |
| Goat Anti-Rabbit IgG antibody | Abbkine | Cat#.A23620 |
| Goat Anti-Mouse IgG antibody | Abbkine | Cat#.A23210 |
| Anti-Flag-tag antibody | Proteintech | Cat#20543-1-AP |
| Anti-HA-tag antibody | MBL | Cat#M180-11 |
| The shRNA primers of MRPL3 |  |  |
| sh#1 | 5′-AATGTAATAGAAGGCGCACCG-3′ | HANBIO |
| sh#2 | 5′-GCUACAUCCAUAUUGGAAUTT-3′ | HANBIO |
| The siRNA primers of TOMM40 and TFAM |  |  |
| TOMM40 siRNA | 5′-GCACGCAACAUACUACCACTT-3′ | HANBIO |
| TFAM siRNA | 5′-CAGCUUGCUAAAGAUGAUATT-3′ | HANBIO |
| The primer sequences of qRT-PCR |  |  |
| Human-*GAPDH*-F | AGGTGAAGGTCGGAGTCAACG | Generay |
| Human-*GAPDH*-R | AGGGGTCATTGATGGCAACA | Generay |
| Human-*MRPL3*-F | TGCTGCTCACTTTCGTCCA | Generay |
| Human-*MRPL3*-R | TCGTTTGACCATGCGTAGC | Generay |
| Human-*TOMM40*-F | CTCTGTGGATAGCAACTGGATCGTG | Sangon |
| Human-*TOMM40*-R | TGTTCTTGCGGTGATTCAGGAAGG | Sangon |
