# Supplementary material for: MRPL3 enhances mitochondrial function via the TOMM40/PGC-1α/TFAM axis to drive early recurrence in hepatocellular carcinoma
Source: Cell Death Dis. 2026 Jun 9;17(1):721. doi: 10.1038/s41419-026-08968-8 (PMC13469937; doi:10.1038/s41419-026-08968-8)
Supplement: Supplementary file 3 [file 41419_2026_8968_MOESM11_ESM.docx]

**Table s3. Baseline Characteristics of the Study Population**

| Characteristics | Low expression | High expression | P value |
| --- | --- | --- | --- |
| n | 43 | 97 |  |
| Gender, n (%) |  |  | 0.975 |
| Male | 36 (83.7%) | 81 (83.5%) |  |
| Female | 7 (16.3%) | 16 (16.5%) |  |
| Age (years), median (IQR) | 58 (51, 66) | 60 (54, 64) | 0.473 |
| Hepatitis (Yes/No), n (%) |  |  | 0.245 |
| 1 | 35 (81.4%) | 70 (72.2%) |  |
| 0 | 8 (18.6%) | 27 (27.8%) |  |
| Cirrhosis (Yes/No), n (%) |  |  | 0.200 |
| 1 | 35 (81.4%) | 69 (71.1%) |  |
| 0 | 8 (18.6%) | 28 (28.9%) |  |
| AFP, median (IQR) | 8.84 (5.195, 565.85) | 70.33 (5.03, 426.9) | 0.285 |
| Maximum tumor diameter (≥5 cm = 1, <5 cm = 0), n (%) |  |  | 0.940 |
| 1 | 17 (39.5%) | 39 (40.2%) |  |
| 0 | 26 (60.5%) | 58 (59.8%) |  |
| MVI ( Yes/No), n (%) |  |  | 0.030 |
| 1 | 4 (9.3%) | 22 (22.7%) |  |
| 0 | 39 (90.7%) | 75 (77.3%) |  |
| AJCC 8th edition stage（Ⅰ-Ⅱor Ⅲ-Ⅳ）, n (%) |  |  | 0.513 |
| 3 | 9 (20.9%) | 13 (13.4%) |  |
| 2 | 8 (18.6%) | 18 (18.6%) |  |
| 1 | 26 (60.5%) | 66 (68%) |  |
| BCLC stage（0-A or B-C）, n (%) |  |  | 0.383 |
| B | 6 (14%) | 12 (12.4%) |  |
| A | 34 (79.1%) | 70 (72.2%) |  |
| C | 3 (7%) | 15 (15.5%) |  |
