# Supplementary material for: MRPL3 enhances mitochondrial function via the TOMM40/PGC-1α/TFAM axis to drive early recurrence in hepatocellular carcinoma
Source: Cell Death Dis. 2026 Jun 9;17(1):721. doi: 10.1038/s41419-026-08968-8 (PMC13469937; doi:10.1038/s41419-026-08968-8)
Supplement: Supplementary file 4 [file 41419_2026_8968_MOESM12_ESM.docx]

| Characteristics | Total(N) | Univariate analysis | |  | Multivariate analysis | |
| --- | --- | --- | --- | --- | --- | --- |
|  |  | Hazard ratio (95% CI) | P value |  | Hazard ratio (95% CI) | P value |
| Gender | 140 |  |  |  |  |  |
| Male | 117 | Reference |  |  |  |  |
| Female | 23 | 0.671 (0.374–1.206) | 0.182 |  |  |  |
| Age (years) | 140 | 1.012 (0.990–1.036) | 0.291 |  |  |  |
| Expression of MRPL3 | 140 | 1.136 (1.059–1.218) | **<0.001*** |  | 1.146 (1.068–1.229) | **<0.001*** |
| Hepatitis (Yes/No) | 140 |  |  |  |  |  |
| 1 | 105 | Reference |  |  |  |  |
| 0 | 35 | 0.871 (0.541–1.404) | 0.571 |  |  |  |
| Cirrhosis (Yes/No) | 140 |  |  |  |  |  |
| 1 | 104 | Reference |  |  |  |  |
| 0 | 36 | 0.910 (0.569–1.456) | 0.696 |  |  |  |
| AFP | 140 | 1.000 (1.000–1.000) | 0.920 |  |  |  |
| Maximum tumor diameter (≥5 cm = 1, <5 cm = 0) | 140 |  |  |  |  |  |
| 0 | 84 | Reference |  |  |  |  |
| 1 | 56 | 1.170 (0.779–1.756) | 0.449 |  |  |  |
| MVI ( Yes /No) | 140 |  |  |  |  |  |
| 0 | 114 | Reference |  |  | Reference |  |
| 1 | 26 | 2.160 (1.350–3.456) | **0.001*** |  | 1.451 (0.876–2.403) | 0.148 |
| AJCC 8th edition stage | 140 |  |  |  |  |  |
| 3 | 22 | Reference |  |  | Reference |  |
| 2 | 26 | 0.919 (0.496–1.701) | 0.788 |  | 0.957 (0.515–1.780) | 0.889 |
| 1 | 92 | 0.446 (0.264–0.753) | **0.003*** |  | 0.434 (0.248–0.759) | **0.003*** |

**Table s4. Univariate and multivariate Cox regression analyses for DFS**
