# Supplementary material for: MRPL3 enhances mitochondrial function via the TOMM40/PGC-1α/TFAM axis to drive early recurrence in hepatocellular carcinoma
Source: Cell Death Dis. 2026 Jun 9;17(1):721. doi: 10.1038/s41419-026-08968-8 (PMC13469937; doi:10.1038/s41419-026-08968-8)
Supplement: Supplementary file 5 [file 41419_2026_8968_MOESM13_ESM.docx]

| Characteristics | Total(N) | Univariate analysis | |  | Multivariate analysis | |
| --- | --- | --- | --- | --- | --- | --- |
|  |  | Hazard ratio (95% CI) | P value |  | Hazard ratio (95% CI) | P value |
| Gender | 140 |  |  |  |  |  |
| Male | 117 | Reference |  |  |  |  |
| Female | 23 | 0.998 (0.523–1.904) | 0.995 |  |  |  |
| Age (years) | 140 | 1.019 (0.991–1.047) | 0.196 |  |  |  |
| Expression of MRPL3 | 140 | 1.269 (1.158–1.390) | **<0.001*** |  | 1.273 (1.161–1.395) | **<0.001*** |
| Hepatitis (Yes/No) | 140 |  |  |  |  |  |
| 1 | 105 | Reference |  |  |  |  |
| 0 | 35 | 1.572 (0.932–2.650) | 0.090 |  |  |  |
| Cirrhosis (Yes/No) | 140 |  |  |  |  |  |
| 1 | 104 | Reference |  |  | Reference |  |
| 0 | 36 | 1.648 (0.984–2.759) | 0.057 |  | 1.226 (0.720–2.087) | 0.452 |
| AFP | 140 | 1.000 (1.000–1.000) | 0.507 |  |  |  |
| Maximum tumor diameter (≥5 cm = 1, <5 cm = 0) | 140 |  |  |  |  |  |
| 0 | 84 | Reference |  |  |  |  |
| 1 | 56 | 1.031 (0.634–1.676) | 0.903 |  |  |  |
| MVI ( Yes/No) | 140 |  |  |  |  |  |
| 0 | 114 | Reference |  |  | Reference |  |
| 1 | 26 | 1.543 (0.880–2.705) | 0.130 |  | 0.742 (0.406–1.356) | 0.332 |
| AJCC 8th edition stage | 140 |  |  |  |  |  |
| 3 | 22 | Reference |  |  | Reference |  |
| 2 | 26 | 0.832 (0.427–1.620) | 0.589 |  | 0.736 (0.377–1.438) | 0.370 |
| 1 | 92 | 0.319 (0.175–0.580) | **<0.001*** |  | 0.259 (0.138–0.483) | **<0.001*** |

**Table s5. Univariate and multivariate Cox regression analyses for OS**
