# Supplementary material for: MRPL3 enhances mitochondrial function via the TOMM40/PGC-1α/TFAM axis to drive early recurrence in hepatocellular carcinoma
Source: Cell Death Dis. 2026 Jun 9;17(1):721. doi: 10.1038/s41419-026-08968-8 (PMC13469937; doi:10.1038/s41419-026-08968-8)
Supplement: Supplementary file 6 [file 41419_2026_8968_MOESM14_ESM.docx]

**Supplemental Methods**

**1 LC–MS/MS Analysis**

Tissue samples were thawed at 4 °C and lysed with 10 volumes of protein lysis buffer on ice for 10 min, followed by mechanical homogenization under cold conditions. The lysates were centrifuged, and the supernatants were collected. A total of 40 μg protein was subjected to reduction and alkylation, followed by tryptic digestion overnight at 37 °C using the filter-aided sample preparation (FASP) method. The resulting peptides were vacuum-dried and stored at −80 °C until analysis.

Peptides were separated using an EASY-nLC 1200 nano-liquid chromatography system (Thermo Fisher Scientific) and analyzed on a Q Exactive HF-X mass spectrometer (Thermo Fisher Scientific). The mobile phase consisted of 0.1% formic acid in water (solvent A) and 0.1% formic acid in acetonitrile (80%) (solvent B). Peptides were first loaded onto a trap column (2 cm × 100 μm, C18) and then separated on an analytical column (30 cm × 150 μm, C18) at a flow rate of 600 nL/min. The elution gradient increased from 6% to 100% solvent B. Spectrometry was performed in positive ion mode with a full MS scan range of 300–1400 m/z. The MS1 resolution was set to 120,000 at m/z 200, with an automatic gain control (AGC) target of 3 × 10⁶ and a maximum injection time of 30 ms. The dynamic exclusion time was set to 12 s. For data-dependent acquisition, the top 60 most intense precursor ions were selected for fragmentation by higher-energy collisional dissociation (HCD) with a normalized collision energy (NCE) of 27%. The MS/MS resolution was set to 7,500. Raw MS data were processed using Proteome Discoverer for database searching and quantitative analysis. Trypsin was specified as the digestion enzyme with up to two missed cleavages allowed. Carbamidomethylation of cysteine was set as a fixed modification, while oxidation of methionine and acetylation of protein N-termini were set as variable modifications. The precursor mass tolerance was set to 20 ppm and the fragment mass tolerance to 50 mmu. The database used for protein identification was the NCBI Human protein database.

**2 Extracellular Acidification Rate (ECAR) Measurement**

Extracellular acidification rate (ECAR) was determined with a Seahorse XF96 Extracellular Flux Analyzer (Agilent Seahorse Bioscience) following protocols recommended by the manufacturer. Cells were seeded on XF96 cell culture plates (10,000 cells/well) except the background correction wells. After attachment overnight under normal culture conditions, cells were transferred to a non-CO₂ incubator for 30 min in non-buffered assay medium (Agilent) before the assay. Baseline measurements were recorded, followed by sequential injection of 10 mM glucose, 1 μM oligomycin, and 50 mM 2-deoxy-D-glucose (2-DG). The assay was performed using the XF Glycolysis Stress Test Kit (Agilent).

**3 RNA-seq library preparation and bioinformatic analysis**

Total RNA was extracted using TRIzol® reagent (Invitrogen, USA) following the manufacturer’s instructions. RNA purity and concentration were assessed using a NanoDrop ND-2000 spectrophotometer (Thermo Scientific, USA) based on the A260/A280 ratio, and RNA integrity was evaluated using an Agilent Bioanalyzer 4150 system (Agilent Technologies, USA). Only high-quality RNA samples were used for library construction.

mRNA libraries were prepared using the ABclonal mRNA-seq Library Preparation Kit (ABclonal, China). Briefly, mRNA was enriched from 1 μg total RNA using oligo(dT) magnetic beads and subsequently fragmented using divalent cations at elevated temperature. First-strand cDNA was synthesized using random hexamer primers and reverse transcriptase, followed by second-strand cDNA synthesis using DNA polymerase I and RNase H. The resulting double-stranded cDNA fragments were ligated with sequencing adapters and amplified by PCR. PCR products were purified using the AMPure XP system, and library quality was assessed using an Agilent Bioanalyzer 4150.

Sequencing was performed on an Illumina NovaSeq 6000 / MGISEQ-T7 platform to generate paired-end reads. Raw reads in FASTQ format were processed to remove adapter sequences and low-quality reads. Reads with >60% bases having a quality score ≤25 or with >5% ambiguous bases (N) were discarded to obtain high-quality clean reads. Clean reads were aligned to the human reference genome using HISAT2 (v2.2.1). Gene-level read counts were obtained using featureCounts (v2.0.3), and gene expression levels were normalized as FPKM (Fragments Per Kilobase of transcript per Million mapped reads). Differential expression analysis was performed using DESeq2 (v1.34.0). Genes with |log₂ fold change| ≥ 1 and adjusted P value (FDR) < 0.05 were considered differentially expressed genes (DEGs).

**4 Mitochondrial isolation**

Mitochondria were isolated from cultured HCC cells using a mitochondrial isolation kit (C3601, Beyotime, China) according to the manufacturer’s instructions. All procedures were performed on ice. Cells were homogenized in isolation buffer supplemented with PMSF (1 mM) and subjected to differential centrifugation (1,000×g for 10 min followed by 3,500×g for 10 min at 4 °C) to obtain the mitochondrial pellet. The mitochondria were washed once and lysed for protein analysis. Purity was verified by immunoblotting using mitochondrial and cytosolic markers. For mitochondrial co-immunoprecipitation, isolated mitochondria were lysed in NP-40 buffer containing PMSF.

**5 Cycloheximide (CHX) chase assay**

To assess TOMM40 protein stability, a cycloheximide (CHX) chase assay was performed. HCC cells (vector or MRPL3-overexpressing) were treated with CHX (50 μg/mL) to block de novo protein synthesis. Cells were harvested at 0, 3, 6, and 9 h after CHX addition, lysed in RIPA buffer containing protease inhibitors, and subjected to immunoblotting. TOMM40 band intensities were quantified by densitometry, normalized to β-tubulin, and expressed relative to the 0 h time point.

**6 Proteasome inhibition assay**

To examine the involvement of proteasomal degradation, cells were treated with the proteasome inhibitor MG-132 (5μM) for 6 h. After treatment, cells were collected and lysed for western blot analysis. TOMM40 protein levels were quantified by densitometry and normalized to β-tubulin. Where indicated, MG-132 was compared between vector and MRPL3-overexpressing conditions.
