# Supplementary material for: MRPL3 enhances mitochondrial function via the TOMM40/PGC-1α/TFAM axis to drive early recurrence in hepatocellular carcinoma
Source: Cell Death Dis. 2026 Jun 9;17(1):721. doi: 10.1038/s41419-026-08968-8 (PMC13469937; doi:10.1038/s41419-026-08968-8)
Supplement: Supplementary file 7 [file 41419_2026_8968_MOESM8_ESM.docx]

**The list of supplementary figure legends**

**Supplementary Figure 1. Proteomic characteristics of HCC samples and prognostic performance of MRPL3-based models**

(A) Number of proteins detected across 15 samples from 12 patients with HCC. (B) Principal component analysis (PCA) of the proteomic profiles. (C) Density distribution of proteomic intensities after log10 transformation. (D–F) (D) LASSO coefficient trajectories identify MRPL3 expression and AJCC stage as prognostic factors for DFS; (E) a nomogram built on these variables, and (F) 1-, 3-, and 5-year calibration plots show good agreement and predictive accuracy. (G–I) (G) LASSO coefficient trajectories identify MRPL3 expression and AJCC stage as prognostic factors for OS; (H) the corresponding nomogram and (I) 1-, 3-, and 5-year calibration plots confirm robust model performance. HCC, hepatocellular carcinoma; LASSO, Least Absolute Shrinkage and Selection Operator.

**Supplementary Figure 2. MRPL3 promotes HCC cell proliferation, migration, and tumor growth**

(A, B) Compared with that of normal hepatocytes, MRPL3 expression is significantly elevated in HCC cell lines at the mRNA level (A, by qRT-PCR) and protein level (B, by Western blot). (C) Wound-healing assay assessing the migratory capacity of the indicated HCC cells, with representative images and quantification. (D) Representative images of excised subcutaneous xenografts at day 30. (E) Tumor volumes measured every 3 days and plotted as growth curves. (F) Comparison of endpoint tumor weights. (G) Representative IHC staining of Ki-67 (scale bar: 50 μm) with quantification. Data are presented as the mean ± SD (n ≥ 3). Significance: ns, not significant; *P < 0.05; **P < 0.01; ***P < 0.001. HCC, hepatocellular carcinoma; qRT-PCR, quantitative reverse transcription polymerase chain reaction; SD, standard deviation.

**Supplementary Figure 3. MRPL3 regulates mitochondrial respiration, glycolysis, and mitochondrial respiratory chain-related protein expression in HCC cells**

(A, B) High-resolution respirometry in HuH-7 (A) and Mahlavu (B) cells following MRPL3 knockdown or overexpression. Representative oxygen consumption rate (OCR) traces under basal conditions and after sequential injections of oligomycin, the uncoupler FCCP, and rotenone + antimycin A are shown. (C, D) Quantification of spare respiratory capacity (SRC) and proton leak in HuH-7 and Mahlavu cells upon MRPL3 knockdown (C) or overexpression (D). (E, F) Seahorse XF Glycolysis Stress Test was performed in Mahlavu (E) and HuH-7 (F) cells. Left panels show extracellular acidification rate (ECAR) traces following sequential injection of glucose, oligomycin, and 2-deoxy-D-glucose (2-DG). Right panels show quantification of glycolysis, glycolytic capacity, and glycolytic reserve. (G, H) Western blot analysis of MRPL3, ND1 (Complex I), and COX I (Complex IV) protein levels in Mahlavu (G) and HuH-7 (H) cells following MRPL3 knockdown. Data are presented as the mean ± SD (n ≥ 3). Significance: ns, not significant; * P < 0.05; ** P < 0.01; *** P < 0.001. HCC, hepatocellular carcinoma; SD, standard deviation.

**Supplementary Figure 4. MRPL3 regulates the PGC-1α/TFAM axis and mitochondrial respiratory function in HCC cells**

(A, B) Bar plots showing PGC-1α and TFAM protein levels following MRPL3 knockdown or overexpression. (C, D)Proton leak and reserve capacity quantification in HCC cells overexpressing MRPL3 with concurrent TFAM knockdown. All experiments were performed in HCC cell lines Mahlavu and HuH-7. Data are presented as the mean ± SD (n ≥ 3). Significance: ns, not significant; * P < 0.05; ** P < 0.01; *** P < 0.001. HCC, hepatocellular carcinoma; SD, standard deviation.

**Supplementary Figure 5. MRPL3 interacts with TOMM40 within mitochondria and enhances TOMM40 protein stability**

(A) Immunoprecipitation coupled with mass spectrometry identified the interacting peptides of MRPL3 and TOMM40. (B) After isolation and purification of mitochondrial fractions from Mahlavu cells, Western blotting was performed to detect the expression of MRPL3, TOMM40, TOMM20, and β-Tubulin. (C) Co-IP was performed to examine the interaction between MRPL3 and TOMM40 in mitochondrial proteins isolated from Mahlavu cells. (D, E) qRT-PCR was performed to detect the mRNA levels of TOMM40 in Mahlavu (D) and HuH-7 (E) cells following MRPL3 knockdown or overexpression. (F, G) Cycloheximide (CHX) chase assays were performed in Mahlavu (F) and HuH-7 (G) cells to assess the effect of MRPL3 overexpression on TOMM40 protein stability, with quantification shown. (H, I) TOMM40 protein levels were detected and quantified in Mahlavu (H) and HuH-7 (I) cells after MG-132 treatment to evaluate the effect of MRPL3 on TOMM40 protein degradation. Data are presented as the mean ± SD (n ≥ 3). ns, not significant; *P < 0.05; **P < 0.01; ***P < 0.001.

**Supplementary Figure 6. Quantification of TOMM40 and PGC-1α/TFAM axis-related protein levels regulated by MRPL3**

(A) Bar plot showing TOMM40 protein levels following MRPL3 knockdown or overexpression. (B, C) Bar plots showing PGC-1α and TFAM protein levels with MRPL3 overexpression combined with TOMM40 knockdown. All experiments were performed in HCC cell lines Mahlavu and HuH-7. Data are presented as the mean ± SD (n ≥ 3). Significance: ns, not significant; * P < 0.05; ** P < 0.01; *** P < 0.001. HCC, hepatocellular carcinoma; SD, standard deviation.

**Supplementary Figure 7. TOMM40 is involved in MRPL3-mediated enhancement of mitochondrial respiration and malignant phenotypes in HCC cells**

(A, B) Proton leak and reserve capacity quantification in Mahlavu (A) and HuH-7 (B) cells overexpressing MRPL3 with concurrent TOMM40 knockdown. (C, D) Mahlavu (C) and HuH-7 (D) cells: CCK-8 assays showing proliferation upon MRPL3 overexpression with concurrent TOMM40 knockdown. (E) Mahlavu and HuH-7 cells: representative images and quantification of colony formation under MRPL3 overexpression with TOMM40 knockdown. (F, G) Mahlavu and HuH-7 cells: Transwell migration (F) and invasion (G) assays (scale bar: 100 μm) Data are presented as the mean ± SD (n ≥ 3). Significance: ns, not significant; * P < 0.05; ** P < 0.01; *** P < 0.001. HCC, hepatocellular carcinoma; SD, standard deviation.
